# Clustering of Death Receptor for Apoptosis Using Nanoscale Patterns of Peptides

**DOI:** 10.1021/acsnano.0c10104

**Published:** 2021-05-21

**Authors:** Yang Wang, Igor Baars, Ferenc Fördös, Björn Högberg

**Affiliations:** Department of Medical Biochemistry and Biophysics, Karolinska Institutet, SE-17177 Stockholm, Sweden

**Keywords:** TRAIL-mimicking peptide, death receptor clustering, DNA origami, hexagonal pattern, apoptosis

## Abstract

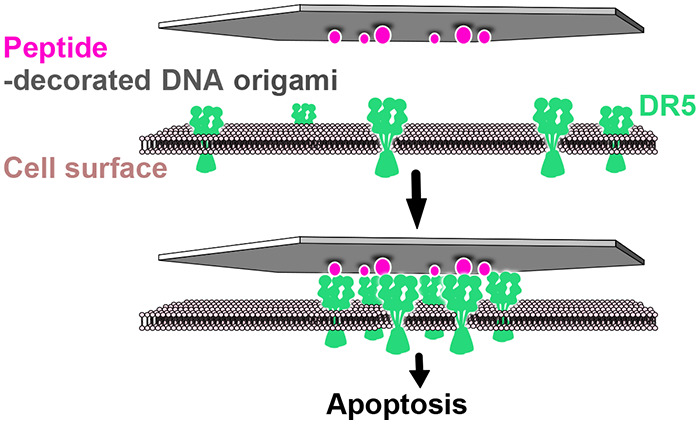

The nanoscale spatial organization of transmembrane tumor necrosis factor (TNF) receptors has been implicated in the regulation of cellular fate. Accordingly, molecular tools that can induce specific arrangements of these receptors on cell surfaces would give us an opportunity to study these effects in detail. To achieve this, we introduce DNA origami nanostructures that precisely scaffold the patterning of TNF-related apoptosis-inducing ligand-mimicking peptides at nanoscale level. Stimulating human breast cancer cells with these patterns, we find that around 5 nm is the critical interligand distance of hexagonally patterned peptides to induce death receptor clustering and a resulting apoptosis. We thus offer a strategy to reverse the non-efficacy of current ligand- and antibody-based methods for TNF superfamily activation.

## Introduction

Because the tumor necrosis factor (TNF) receptor superfamily (TNFRSF) plays important roles in cell proliferation, cell death, immune regulation, and morphogenesis, it has been extensively targeted for disease treatment.^1−5^ Although structural information on TNFRSF, corresponding ligands, and even the receptor–ligand complexes have been quite thoroughly characterized,^6,7^ molecular tools and drugs that can effectively trigger TNFRSF signaling are still missing.^3,5^ Currently available agonists usually fail to work as expected.^8^ A typical example is the TNF-related apoptosis-inducing ligand (TRAIL). TRAIL can recognize and bind to death receptor 4 (DR4) and death receptor 5 (DR5).^9^ Human TRAIL (Dulanermin) and DR4/5 agonistic monoclonal antibodies (*e.g.*, Mapatumumab and Lexatumumab) have been under clinical trials as anticancer therapeutics since the mid-1990s.^10,11^ Randomized control trials have however recently shown that their efficiency in terms of survival benefits is lacking.^12,13^ Studies of TRAIL–DR5 complexes on cells displaying apoptosis have revealed that the transmembrane domain of DR5 formed higher-order structures through clustering.^14^ Thus, one potential reason for the lack of efficacy could be that death receptor triggering *via* the natural ligand/antibody–receptor binding mechanism, might not be strong enough to cause receptor clustering. To explore this, anti-TRAIL antibodies, which can cross-link TRAIL, were used together with TRAIL to promote the formation of DR5 clusters.^15,16^ This strategy improved the apoptosis of cancer cells. Another strategy was to covalently multimerize TRAIL or TRAIL-mimicking peptide on peptide-, dextran-, or graphene-based scaffolds,^17−20^ which was also demonstrated to be efficient. However, using these strategies it is typically not possible to precisely control the nanoscale spatial presentation of the proteins or peptides. By conjugating ligands onto surfaces with prepatterned nanodot arrays, other groups have achieved a nanoscale arrangement of TNF with spacings between 58 and 290 nm.^21^ Cell culture on those surfaces showed a dependence on interligand distances, revealing the importance of interligand distance control for efficient death receptor activation. Nevertheless, the achievable smallest interdot distance was the size of nanodots themselves, making, for example, sub-10 nm interdot arrangement a challenge. Also, note that in display experiments where a surface is covered in ligands, both the number and spatial separation between them vary at the same time. Consequently, surfaces with differently spaced ligands would also display different overall ligand amounts at the surface–cell interface, which could also affect cell activity, giving potentially confounded interpretations. On top of this, clinical translations of surface patterning methods are typically limited as the path from patterning large planar surfaces to patterning biocompatible nanoparticles is not straightforward.

In contrast, DNA origami nanostructures^22−29^ offer both a programmable way to precisely display biomolecule nanopatterns from monodisperse particles and, through this, a way to display these to cells from the solution phase. This allows us to vary the separation of ligands independently from the total dosage or total concentration of ligands and further allows us to focus solely on the spatial separation between ligands on the nanoscale. Thanks to its spatial addressability,^30−33^ varying nanopatterns of ephrin-A5,^34,35^ caspase-9 variant,^36^ antigens of human IgGs and IgMs,^37^ immunogen eOD-GT8,^38^ and Fas ligands^39^ on DNA origami nanostructures have been studied, showing an increasing importance for biomedical applications.

## Results and Discussion

**Figure 1:**
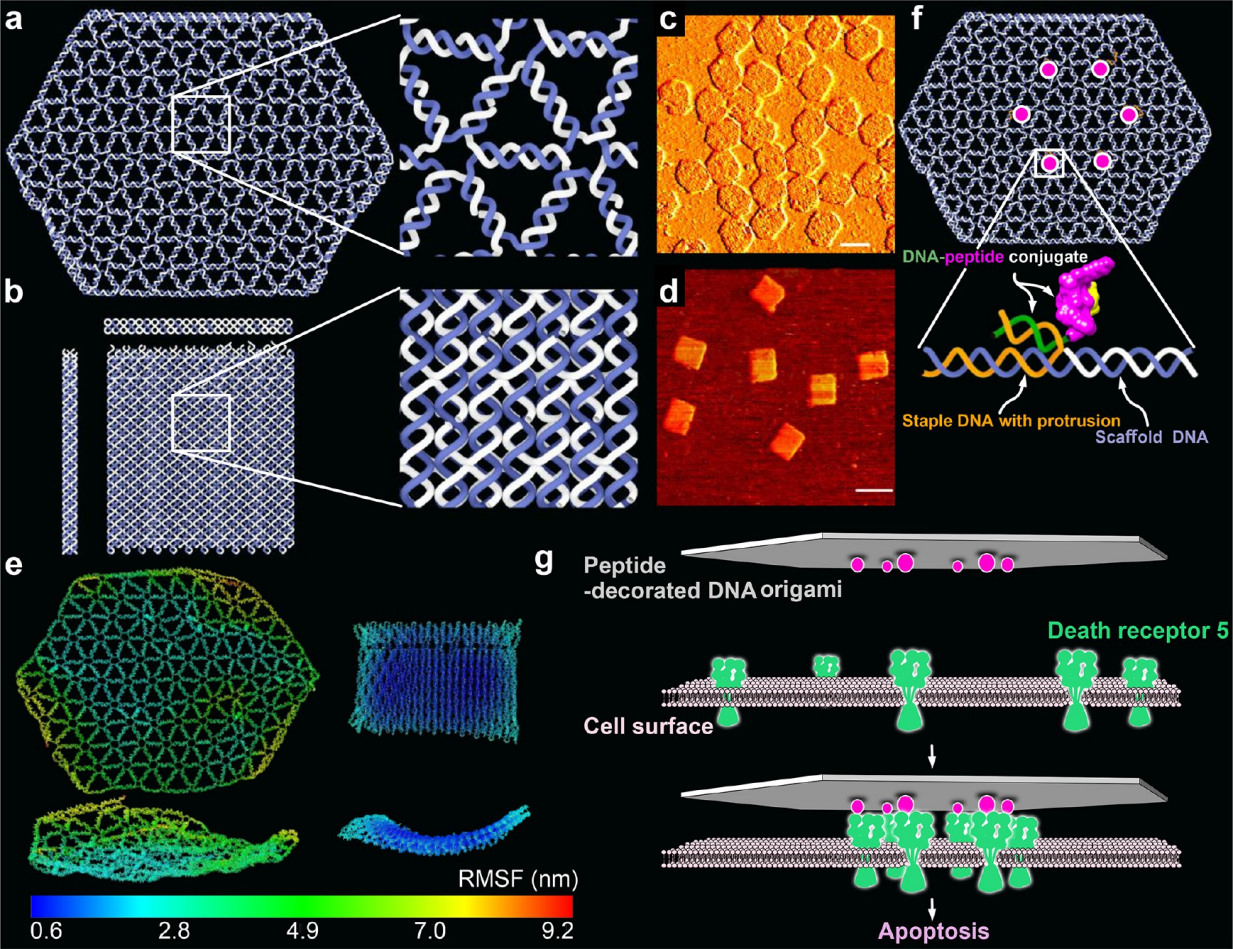
Structure design and characterization. (a) Stylized renderings of the single-layer W-structure (top view), together with a highlight of its DNA arrangement details. (b) Stylized renderings of the double-layer L-structure (side view, front view, and top view), together with a highlight of its DNA arrangement details. The scaffold DNA is colored in dark blue, and staples are in gray. (c and d) Two types of structures imaged by atomic force microscopy. Scale bars are 100 nm. (e) Computed mean structures and RMSF of the W- and L-structures using oxDNA, top and front views. (f) Schematic illustration of how the TRAIL-mimicking peptides are attached to the DNA origamis (same scheme in both W and L). (g) Schematic illustration of how breast cancer cell apoptosis can be triggered by peptide patterns on DNA origami templates by inducing clustering of death receptor 5.

To investigate the effect of differing ligand pattern sizes on death receptors, we prepared two versions of DNA origami as templates: a single-layer wire frame (W) flat sheet (Figure 1a) and a double-layer square lattice (L) style flat sheet (Figure 1b and Supporting Information (SI) Figures S1 and S2). Sharp electrophoresis bands of structures (before and after purification) on 2% agarose gels (Figure S3) and expected structural characteristics under atomic force microscopy (AFM) (Figure 1c,d) indicate successful structure preparations. We produced a collection of structures displaying protruding 5′ single-stranded DNA (ssDNA) handles for subsequent hybridization of ssDNA–ligand conjugates.

Because of the different structural design methods for W and L, they are expected to possess differences in local rigidity and propensity for thermal fluctuation. The mean structures, which were computed from oxDNA molecular dynamics simulation^40,41^ in 500 mM Na^+^ (simulation parameter), showed that the L-type structure tended to stay flatter than the W-types (Figure 1e). Root mean square fluctuation (RMSF) values of the Ws are estimated to be around 5 times higher than those of the Ls (Figure 1e), further validating its higher local structural flexibility. These differences are a consequence of the design schemes that can be attributed to the fact that DNA helices in L (Figure 1b) are more restrained through multiple connections to other helices in the structures as is common in lattice-based 3D origami. This analysis and similar previous investigations on wire frame structures^42^ suggest that the displayed separations on the W-structures are probably fluctuating more during experiments than the corresponding ones on the L-structures.

Homotrimeric TRAIL is the natural ligand of DR5, although it is still not clear if the oligomeric state of the preligand DR5 association is trimeric (Figure S7a)^43^ or dimeric (Figure S7e).^7,14,44^ We used a small cyclic peptide composed of 17 amino acids (Figure S5). This peptide has shown its ability to compete the binding of homotrimeric TRAIL to preligand DR5 association and thus mimic TRAIL’s functions.^45,46^ The rationale for this was 2-fold: (1) The much smaller spatial dimension of the peptide, which has its van der Waals radius at ∼1.5 nm (Figure S4a), could allow sub-10 nm interligand spacing, while the spatial dimension of homotrimeric TRAIL (PDB ID 1DG6) itself is already around 10 nm (Figure S4b). (2) There is an ease of DNA conjugation: conjugating a protein such as TRAIL monomer/trimer with ssDNA could potentially result in multiple modifications per protein or complex. This would in turn risk having one protein occupy more than one binding site of the DNA origami, impeding the formation of expected protein patterns. In contrast, we could easily achieve one ssDNA per peptide conjugation by chemically targeting one prefunctionalized site of the peptide.

We produced the ssDNA–peptide conjugate *via* click chemistry between an azide functionalized lysine residue located at the C-terminal of the peptide and a dibenzocyclooctyne (DBCO) modification at the 5-prime end of the ssDNA (Figure S5). On native polyacrylamide gels, the conjugate had a slower electrophoretic migration than the peptide itself, which was clearly visualized by fluorescent DNA labeling and peptide staining (Figure S6), verifying the reliability of this conjugation method. Importantly, we used a strategy where the conjugation site is on the 5-prime end and binding to a 5-prime end protrusion, thus constraining the peptide close to the site on the DNA origami where the protrusion originates (Figure 1f). Proceeding this way, we avoid a large distance being introduced by the hybridization.

Previous studies on DR5 clusters on apoptotic cells have led to a hypothesis that a nanoscale hexagonal DR5 network formed through the dimerization of DR5 trimers (Figure S7b,c) can directly result in apoptosis.^47^ More recent studies have then shown that the signaling driver is more likely due to the formation of higher-order transmembrane helix (TMH) structures *via* the trimerization of DR5 dimers (Figure S7f,g). Similar to earlier hypotheses though, the potential clustering network mediated by DR5 TMH would again be presented by a hexagonal pattern.^14^ This formation, however, is inhibited by the extracellular domain of DR5 unless the receptor is externally induced to cluster. Antibody AMG655 can promote the homotrimeric TRAIL’s efficacies on DR5 clustering and antitumor activity. Position modeling of the crystallographically decoded TRAIL-DR5-AMG 655 Fab ternary complex further emphasized the importance of the hexagonal honeycomb DR5 pattern on apoptosis (Figure S7d,h).^15^ Following this, we aimed to develop a method to precisely induce this active hexagonally organized DR5 network in breast cancer cells, irrespective of whether the actual molecular mechanism to trigger apoptosis is dimerization of DR5 trimers or trimerization of DR5 dimers (Figure 1g).

**Figure 2:**
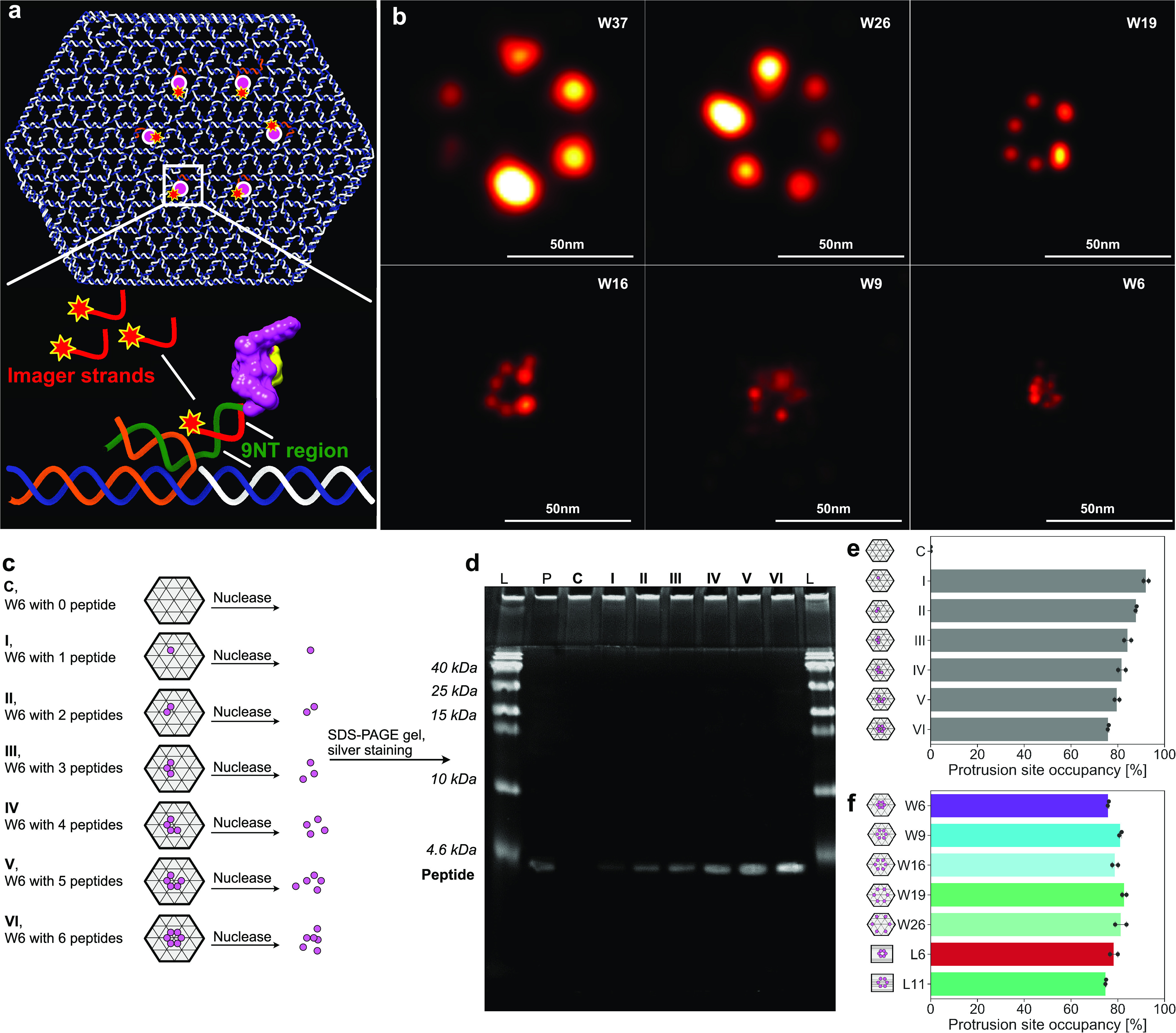
Peptide pattern imaging and quantification. (a) Schematic illustration of how Atto 550-labeled imager strands transiently bind to the 9 extra nucleotides (NTs) region between the peptide and the origami-hybridizing region of the DNA in the ssDNA–peptide conjugate in DNA PAINT experiment. (b) Representative DNA-PAINT images of the differently sized peptide patterns on DNA origamis. Scale bars are 50 nm. W37 is a 37 nm peptide pattern on a wire frame structure used exclusively as a reference during PAINT imaging and analysis. (c and d) W6-structures decorated with varying numbers of peptides were treated with DNase I to completely digest the DNA origami template. The samples were then run on an SDS-PAGE gel, imaged *via* silver staining. L = protein ladder, P = peptide alone, C = empty origami control, I–VI = decorated origamis according to panel c. (e) Dosiometry of the gel images plotted as estimated protrusion site occupancy by ssDNA–peptide conjugate on W6 (with varying numbers of protruding ssDNA sites). (f) Similar analysis of all estimated calculated protrusion site occupancies by ssDNA–peptide conjugate on the different DNA origamis (with 6 protruding DNA sites) used in the study.

We thus decided to investigate patterns of peptides displayed in hexagons, where all patterns had sizes less than 50 nm in intrapeptide spacing. We investigated 5.7 nm (W6), 9.43 nm (W9), 15.8 nm (W16), 18.8 nm (W19), and 25.5 nm (W26) peptide patterns on the W-structures (Figure S8) and 6.3 nm (L6) and 11.1 nm (L11) peptide patterns on the square L-structures (Figures S9 and S10). The nominal distances used in the naming of the structures are taken from the mean distance of the six individual nucleotide-to-nucleotide distances (corresponding to the 6 edges of the hexagons) on the DNA origami from oxDNA molecular dynamics simulations (Figure S11). First, electrophoresis of these structures on agarose gel shows clean monomeric products (Figures S12 and S13). Peptide attachment does not appear to affect the gel mobility, which could be expected since the molecular weight increase (from 5.02 to 5.06 MDa), resulting from hybridizing the peptide–DNA conjugates to the origamis, is minute (0.80% increase). Instead, to verify the correct localization of peptides on structures, we performed a DNA-PAINT experiment: For this analysis only, we inserted 9 extra nucleotides (NTs) between the peptide and the origami-hybridizing region close to the 5′ of the DNA in the ssDNA–peptide conjugates (Figure 2a). These 9 NT regions were used as docking sites for transient binding of Atto 550-labeled DNA imager strands. This transient binding process was then imaged using DNA-PAINT.^48−50^ Imaging results showed the expected hexagonal patterns (Figure 2b and Figures S14–S23), indicating an overall correct localization of the peptides on DNA origami. Not all structures in each sample showed a 6-spot hexagonal pattern, and the distribution analysis showed individual site occupancy rates of 49–76%, depending on structure (Figure S24). The occupancy rate was however estimated to be higher following gel analysis (see below), and it is likely that the microscopy analysis is underestimating the incorporation possibly due to the 9nt PAINT-imaging sites getting sterically blocked from PAINT–probe binding as they lie sandwiched between the protruding site dsDNA and the peptide itself. The pattern size distributions calculated from DNA-PAINT data appear smaller, particularly for the larger patterns, than the sizes in design and oxDNA simulations (Figure S25). This is to some degree expected as the wire frame structures have a higher sensitivity for global deformations (which would primarily impact the larger patterns) as the structures land on the biotinylated surface during PAINT imaging. Note that this strategy is not imaging empty sites, but importantly we are estimating the actual incorporation of DNA–peptide conjugates by targeting the conjugate strand itself with the PAINT probes (Figure 2a).

To further verify the stoichiometry of peptides on each structure, we performed a protein gel analysis after a DNase treatment. Briefly, fully assembled and purified test structures including the positioned peptides, were first incubated with DNase I (Figure 2c and Figure S26a). Following this, we ran the resulting degradation products on SDS-PAGE gels and stained for peptides, after which we performed dosimetry analysis on the lanes to estimate the total peptide content in the samples. The analysis showed a clear correlation between the number of protruding DNA sites per structure and the silver staining intensity of peptide bands (Figure 2d and Figure S26b), corroborating a close match between the designed stoichiometry and the experimental implementation of the structures. On both W- and L-structures, we estimate that the average protruding DNA site occupancy by ssDNA–peptide conjugate was decreasing, from around 93% (sample of structure with 1 protrusion site) to around 75% (sample of structure with 6 protrusion sites), with increasing number of protrusion sites (Figure 2e and Figure S26c). This could probably be attributed to a combination of charge- and steric-hindrance effects and is often observed in functionalized DNA origami with many sites.^34,51,52^ In the target hexagonal peptide patterns, the estimated percentage of site occupancy is between 75 and 80% (Figure 2f), which is slightly higher than the corresponding values from the DNA-PAINT assay (Figure S24).

Human cancer cells can respond to TRAIL treatment very differently.^53^ When it comes to breast cancer cells, the triple-negative mesenchymal ones, to which MDA-MB-231 cells belong, were found to be sensitive to TRAIL.^54^ However, those having receptors for estrogen, which include MCF-7 cells, appear to be largely resistant to TRAIL. Those with Her-2 upregulation, including the SK-BR-3 cells, also appear to have a low sensitivity to TRAIL treatment. Although the expression level of death receptors could partially be linked to this phenomenon,^55^ and intracellular negative feedbacks on TRAIL response were revealed in certain cells,^56^ the actual mechanisms behind this discrepancy are still not well understood. We used MDA-MB-231, MCF-7, and SK-BR-3 cell lines to cover all of these three types of human breast cancer.

**Figure 3:**
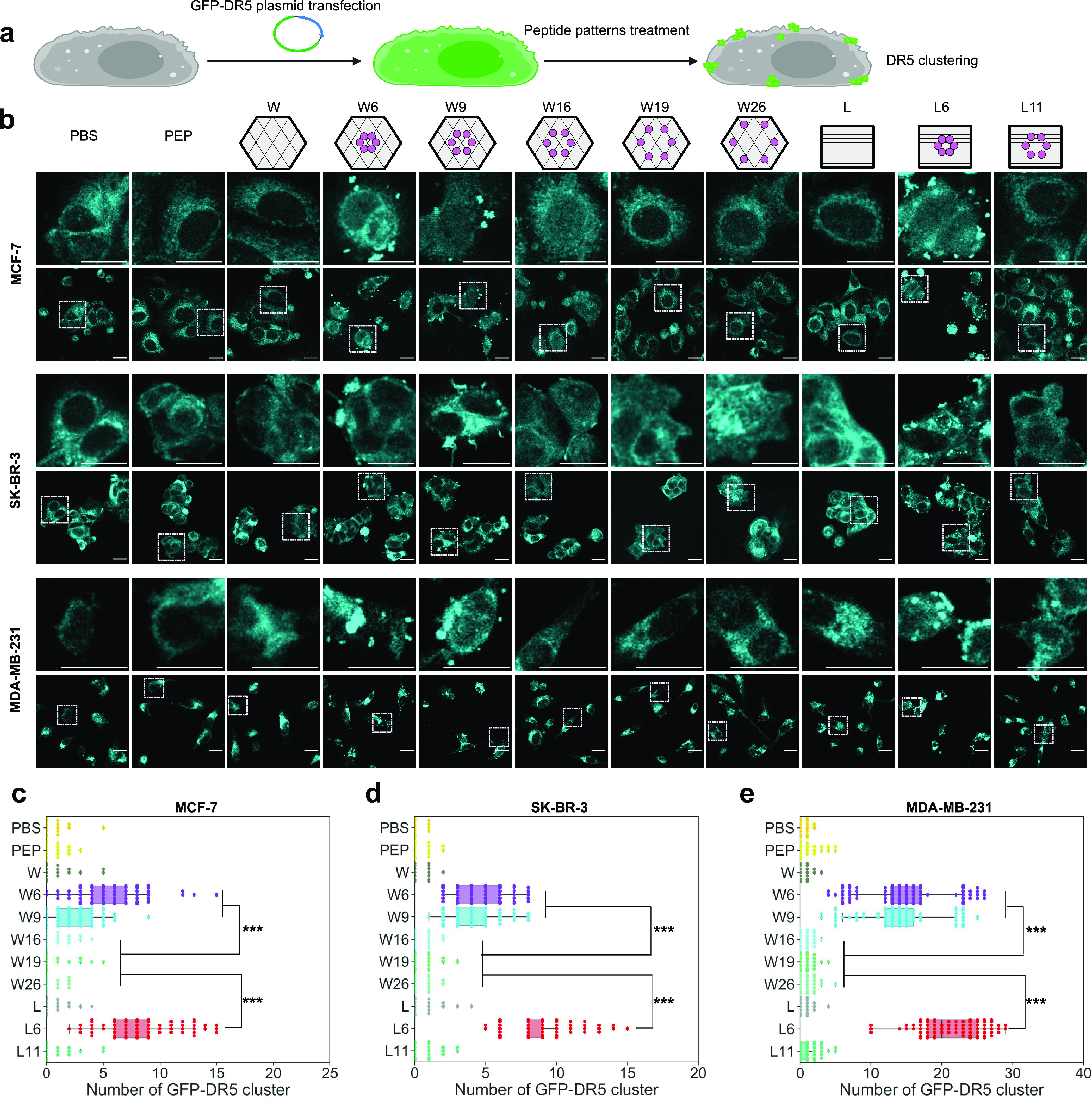
Peptide patterns-induced GFP-DR5 clusters. (a) Experimental workflow. (b) GFP-DR5 (cyan) clusters in breast cancer cells with 4 h treatments of 2-nM DNA origami structures or 12 nM peptides. Scale bars are 20 μm. The enlarged panels above correspond to the indicated areas in the panels below. (c–e) GFP-DR5 cluster counting of MCF-7 cells (c), SK-BR-3 cells (d), and MDA-MB-231 cells (e). Each point stands for the number of GFP-DR5 clusters for one cell (*n* = 100 cells). PBS stands for phosphate-buffered saline, and PEP stands for peptide. ***, *p* < 0.001.

To visualize DR5 clusters, we first established GFP-DR5-expressing cells by plasmid transfection (Figure 3a). We then treated these cells with DNA origami presenting TRAIL-mimicking peptide patterns, following by imaging cells under confocal microscopy. On all cell lines, it showed that (Figure 3b–e) neither did peptide itself nor did DNA origami structures themselves have observable effects on DR5 clustering; for peptide patterns, W6 and W9 successfully caused DR5 clusters, while W16, W19, or W26 did not. This size-dependent effect indicated that, to trigger the process of DR5 clustering, peptides need to be patterned closely enough and around 10 nm seemed to be the critical distance. We also observed significant differences between cells treated with W6 and cells treated with W9. We then further investigated this with the stiffer structures—L6 and L11. The results showed that (Figure 3b–e), being more effective than W6, L6 successfully triggered DR5 clustering. Interestingly, being different from W9, L11 failed to cause DR5 clustering. The main reason for this is probably the different distances (higher spatial density of ligands in W9), but it cannot be ruled out that some of the observed differences could have arisen from the internal structure differences between W- and L-structures, for example a slightly higher charge density of L-structures due to their more closely packed DNA.

**Figure 4:**
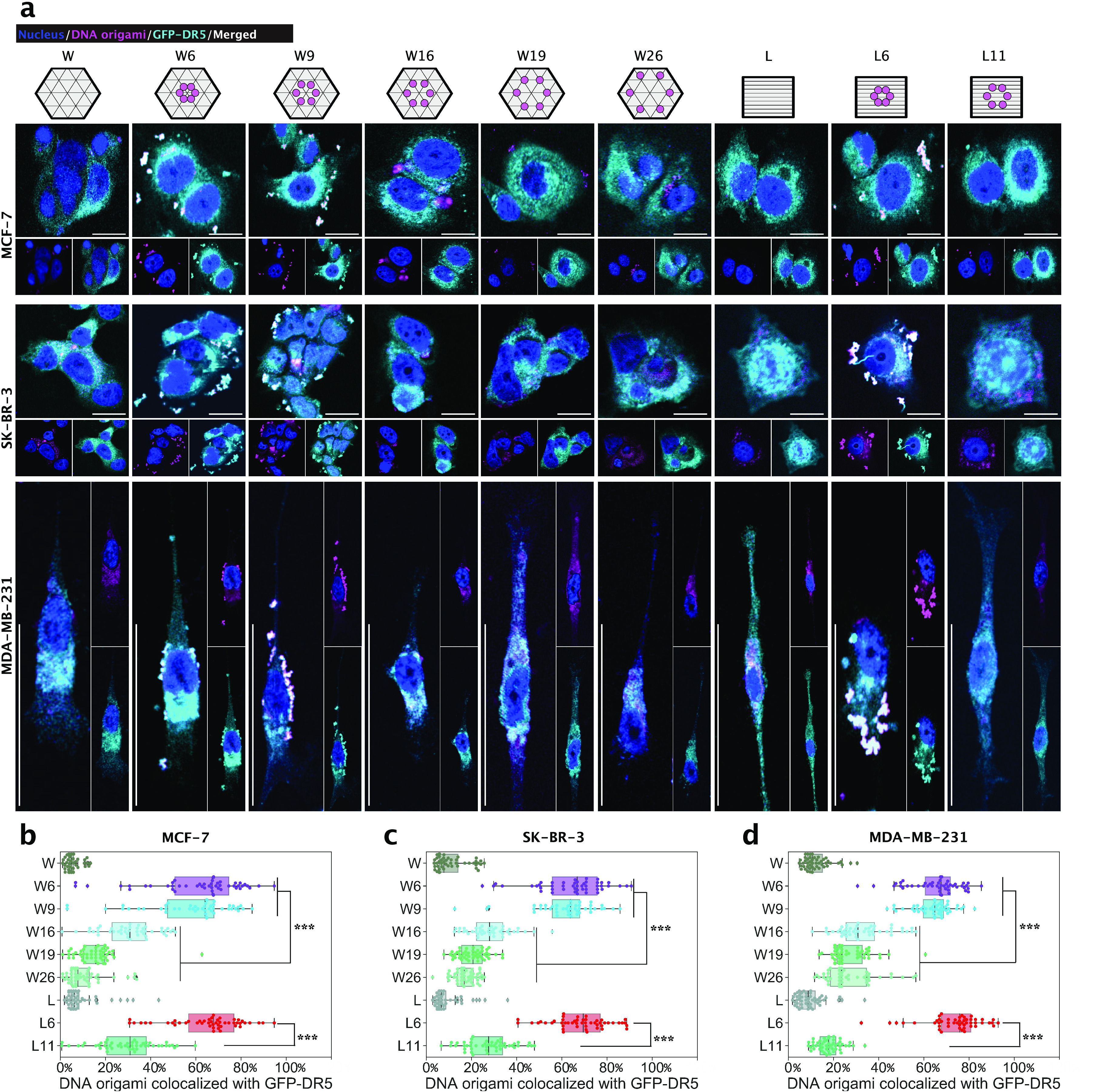
DNA origamis and GFP-DR5 clusters colocalization. (a) Localization of DNA origami (magenta) relative to GFP-DR5 (cyan) clusters. The cells were treated with 2 nM DNA origami structures or 12 nM peptides for 4 h. Scale bars are 20 μm. (b–d) Percentage of DNA origami colocalized with GFP-DR5 clusters (*n* = 50 fields) from MCF-7 (b), SK-BR-3 (c), and MDA-MB-231 (d) cells. ***, *p* < 0.001.

In both W- and L-cases, notably, for all three cell lines, treated cells having GFP-DR5 clusters that showed high colocalizations with Cy5-labeled DNA origami (Figure 4a–d). It shows that, for W6-, W9-, and L6-treated cells, the colocalizations of DNA origami to GFP-DR5 clusters are similarly around 70%. The reasons this is not 100% could be due to (1) Cy5 loss during the washings, (2) the sensitivity limitation of the imaging method, and/or (3) the signal filtering threshold setting during our image analysis. We also found that if we study patterns of L6 with purposely removed peptides, on MCF-7 cells, the extent of DR5 clustering decreased with respect to the removal of peptides (Figure S27), validating the importance of having full patterns for optimal clustering. With these results together, we concluded that DR5 clustering is a precisely controlled cellular process, whose effective triggering needs around 5 nm-spaced hexagonal ligand patterns.

**Figure 5:**
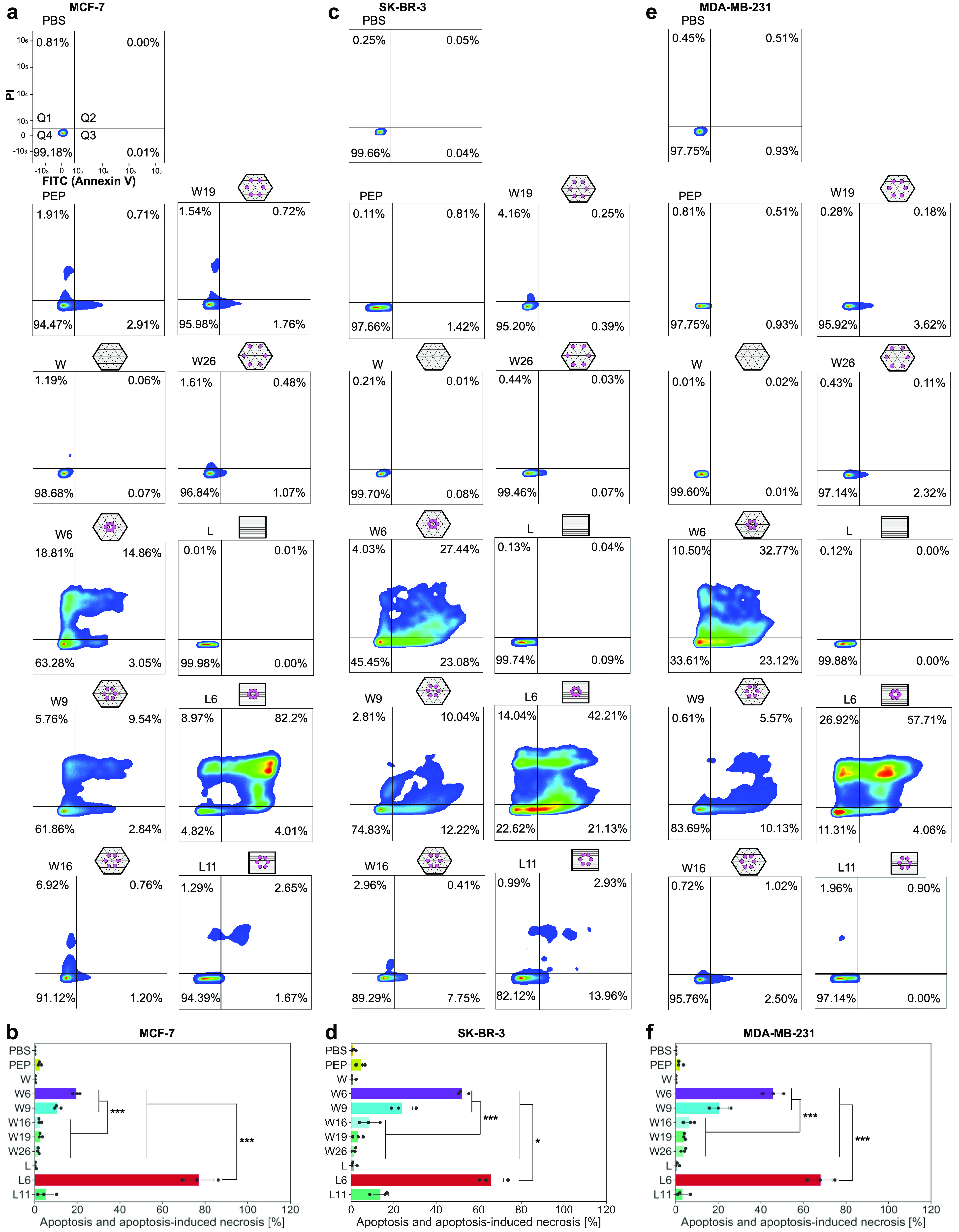
Apoptosis of cells treated with peptide patterns. (a, c, e) Representative MCF-7 (a), SK-BR-3 (c), and MDA-MB-231 (e) cell apoptosis induced by indicated treatments (24 h with 2 nM DNA origami structures or 12 nM peptides). Q1, percentage of cell necrosis; Q2, percentage of apoptosis-induced necrosis; Q3, percentage of cell apoptosis; Q4, percentage of alive cells. (b, d, f) Percentages of apoptosis and apoptosis-induced necrosis (Q2 plus Q3) for MCF-7 (b), SK-BR-3 (d) and MDA-MB-231 (f) cells. Each point stands for the mean value of one biological replicate (*n* = 3). Each biological replicate includes 6 technical replicates. PBS stands for phosphate-buffered saline, and PEP stands for peptide alone. *, *p* < 0.05; ***, *p* < 0.001.

On the basis of these DR5 clustering results, we then measured the apoptosis in nontransfected cell lines. On all three cell lines (Figure 5), W6 or W9 induced significantly more apoptotic and apoptosis-resulted necrotic cells than W16, W19, and W26. For cells treated with L6 or L11, however, only L6 showed a strong apoptosis-inducing capability. Empty DNA origami or peptide itself showed no effect.

**Figure 6:**
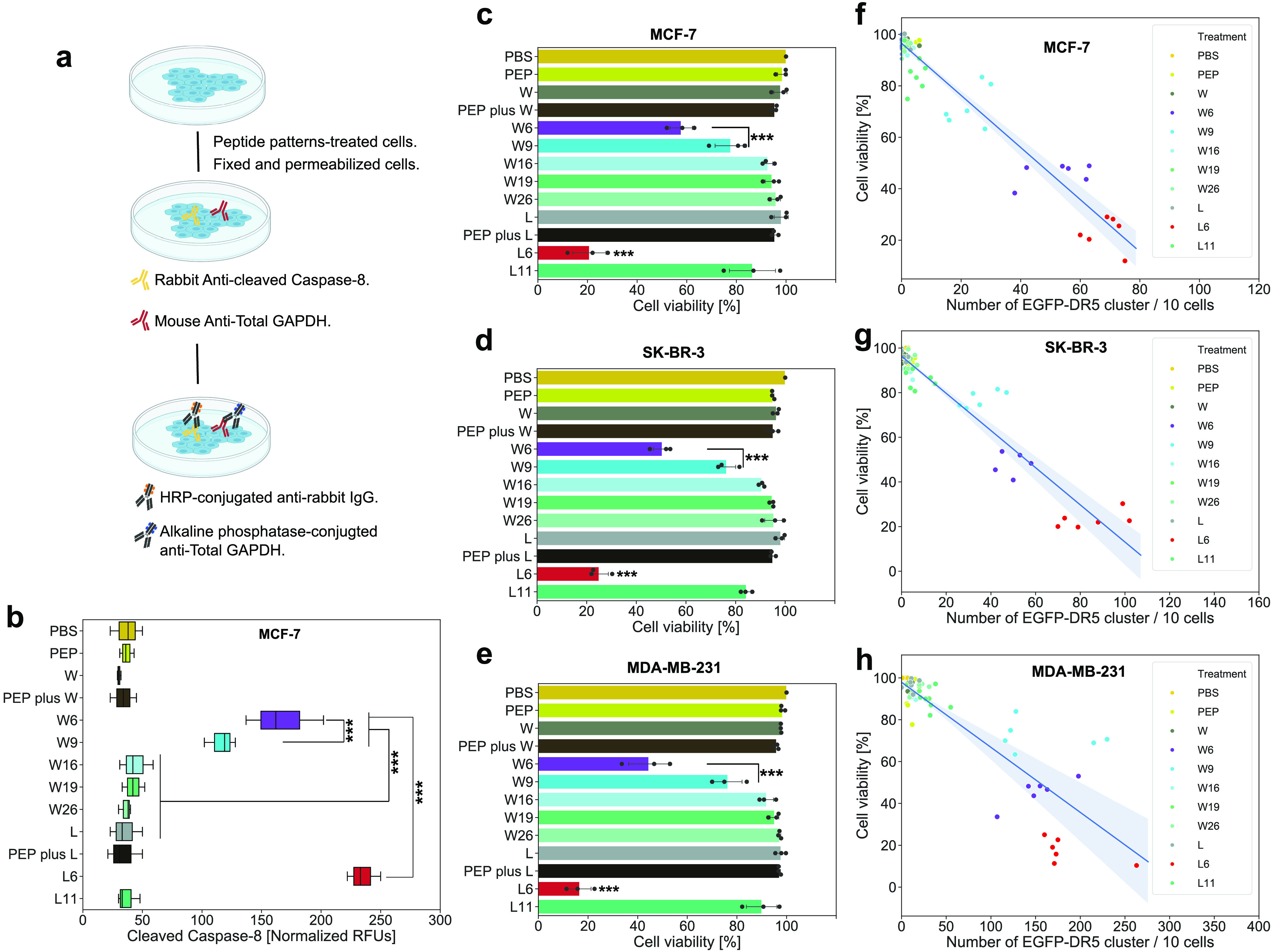
Cell viability. (a) Workflow of cell-based ELISA for cleaved caspase-8 detection. (b) Normalized (to GAPDH) cleaved caspase-8 level after the treatment (1.5 h with 2 nM DNA origami structures or 12 nM peptides). (c–e) Cell viability assay with indicated treatments (48 h with 2 nM DNA origami structures or 12 nM peptides) from MCF-7 (c), SK-BR-3 (d), and MDA-MB-231 (e) cells. Each point stands for the mean of 6 technical replicates from 1 biological replicate. There are 3 biological replicates. (f–h) Correlation of viability of MCF-7 (f), SK-BR-3 (g), and MDA-MB-231 (h) cells to the number (per 10 cells) of GFP-DR5 cluster. For each treatment, 6 groups of independent data are presented. PBS stands for phosphate-buffered saline, and PEP stands for peptide. *, *p* < 0.05; **, *p* < 0.01; ***, *p* < 0.001.

We also checked a cleaved caspase-8 level, which is the key molecular indicator of apoptotic cascades,^57^ by cell-based ELISA (Figure 6a). The most cleaved caspase-8 was detected in MCF-7 cells treated by L6, showing a higher extent of apoptosis and apoptosis-induced necrosis (Figure 6b and Figure S28). On all cell lines, the viability of cells treated by L6 was the lowest among different treatments (Figure 6c–e). This circumvents the previously revealed TRAIL resistance of MCF-7 cell line. The half-maximal inhibitory concentrations (IC_50_) of the peptide were significantly reduced after being patterned on DNA origami (W6, W9, and L6) (Table S1). Combining the data from these measurements and the GFP-DR5 clustering results showed clear correlations (Figure 6f–h), verifying that lower cell viabilities were most likely resulted from an effective DR5 clustering.

To fully determine whether or not these peptide patterns on DNA origami (W6, W9, and L6) are more effective on apoptosis induction than other DR5 agonists, such as cross-linking TRAIL by antibodies^15,16^ or pre-cross-linking TRAIL/TRAIL-mimicking peptides by peptides/proteins/polymers,^17−20^ comparative studies would be needed. Furthermore, the efficacy and potential side effects of our peptide patterns for treatment applications need to be further investigated on animal models.

## Conclusions

In conclusion, we demonstrated an effective strategy of using a DNA origami method to regulate death receptor clustering and following cell apoptosis. This offers a precise and reliable way to understand the importance of nanoscale ligand spatial organization and its control over apoptosis triggering. Notably, this approach does not rely on surface patterning and the stimulating patterns are displayed from solution. The fact that monomeric binders, as were used in this study, are able to trigger apoptosis could indicate that the preligand clustering state of DR5 is less important than was previously thought and that the clustering of DR5, whatever the preligand state may be, is enough to trigger apoptosis. By tuning the size of hexagonal TRAIL-mimicking peptide pattern, we conclude that the interpeptide distance for effective apoptosis was sub-10 nm. Surprisingly, this method worked on both TRAIL-sensitive and breast cancer cells that were previously deemed to be resistant. Our findings also reveal that precise spatial pattern screening of drug molecules at the nanoscale could be a potential way to alleviate some of the non-efficacy problems of currently approved TNFRSF-targeting drugs.

## Experimental Section

### Peptide–DNA Conjugate

We purchased the cyclic peptide (WDCLDNRIGRRQCVKL), with an azide modification at its C-terminal, from JPT Peptide Technologies. We purchased oligonucleotide (TAGATGGAGTGTGGTGTG), with a dibenzocyclooctyne (DBCO) modification at its 5-prime, from Integrated DNA Technologies. The molar ratio of peptide to oligonucleotide in the reaction was 10 to 1. We carried out the reaction in PBS pH 7.4, under room temperature, overnight. We used an Amicon 3K filter tube (Millipore) to purify a small amount (100 μL, 100 nM) of conjugate. The process was done with centrifugation at 14000*g* for 30 min and was repeated 3 times. We purified a big amount of conjugate (above 100 μL and 100 nM) *via* a proFIRE system (Dynamic Biosensors).

### Silver Staining

We performed this using the ProteoSilver Silver Stain Kit (Sigma-Aldrich) according to its established protocol. Briefly, we placed gels in 100 mL of fixing buffer (50 mL of ethanol, 40 mL of Milli-Q water, and 10 mL of glacial acetic acid) overnight. Subsequently, we washed the gels for 10 min in 30% ethanol solution and then in 200 mL of Milli-Q water for 10 min and sensitized the gels in 100 mL of sensitization solution (1 mL of ProteoSilver sensitizer in 99 mL of Milli-Q water). Following this, we washed the gels once more in Milli-Q water for 10 min, equilibrated the gels in silver solution (1 mL of ProteoSilver silver solution and 99 mL of Milli-Q water) for 10 min, followed by two brief washes in Milli-Q water. The gels were then developed for approximately 5 min in 100 mL of developer solution (5 mL of ProteoSilver Developer 1, 0.1 mL of ProteoSilver Developer 2, and 95 mL of Milli-Q water) after which the reaction was stopped by adding 5 mL of the provided stopping solution for at least 10 min. Stained gels were imaged using the GE LAS 4000 gel imager.

### Peptide-Patterned DNA Origami Production

#### Step 1: p8064 Scaffold DNA Production

In a shaker at 37 °C, we cultured *Escherichia coli* (*E. coli*) strain JM109 in 250 mL of 2× Yeast extract Tryptone growth medium (Sigma-Aldrich) with 5 mM MgCl_2_ (Sigma-Aldrich) until its optical density at 600 nm reached 0.5. Then we added the phage containing p8064 scaffold DNA to the bacteria at a multiplicity of infection of 1, after which the phage was amplified with shaking, under 37 °C, for 4 h. We collected the culture and then centrifuged at it 4000*g* for 30 min to pellet the bacteria. The supernatant, which contained the phage, was kept, followed by adding 10 g of PEG8000 (VWR International) and 7.5 g of NaCl (VWR). We then incubated the supernatant with ice for 30 min and centrifuged it at 10000*g* for 30 min to pellet the phage. We resuspended the phage in 10 mL of Tris buffer (10 mM, pH 8.5, VWR) and added 10 mL of a solution containing 0.2 M NaOH (VWR) and 1% SDS. Then we denatured the phage protein coat by adding 7.5 mL of 3 M KOAc (VWR), pH 5.5, and gently mixing, incubating on ice for 10 min. The sample was next centrifuged at 16500*g* for 30 min to pellet the denatured phage proteins. The supernatant containing p8064 scaffold DNA was collected, and 50 mL of 99.5% EtOH (Sigma-Aldrich) was added, mixed gently, and incubated on ice for another 30 min. The sample was centrifuged at 16500*g* for 30 min to pellet the p8064 scaffold DNA. The pellet was then washed with 75% EtOH and air-dried at room temperature for 15 min, followed by resuspending in 10 mM Tris, pH 8.5. The concentration and quality were characterized by UV–vis (NanoDrop, Thermo Scientific) and a 2% agarose gel, respectively.

#### Step 2: DNA Origami Folding and Purification

Staple oligonucleotides (SI Table S1), with the concentration of 100 μM in Milli-Q water, were ordered from Integrated DNA Technologies in 96-well plates. DNA concentrations used for structure folding were as follows: 20 nM ssDNA scaffold and 100 nM per staple DNA. For Cy5-labeled DNA origami, 6 staple DNA strands of the structures were replaced by the same sequences but modified with Cy5 at their 5 primes. All wFS structures were folded in PBS by rapid heat denaturation (80 °C for 5 min) followed by cooling from 80 to 60 °C over 20 min, then from 60 to 24 °C over 14 h. By using the same annealing program, all sFS structures were folded in the buffer containing 13 mM MgCl_2_ (Sigma-Aldrich), 5 mM TRIS (VWR), and 1 mM EDTA (VWR). Folded structures were purified and concentrated by using Amicon 100 K filter tube (Millipore). The process included 6 times washings with the folding buffer at 5000*g* for 2 min.

#### Step 3: Peptide Attachment to DNA Origami

The peptide–DNA conjugates were added with a 10-fold excess to each protruding site on the DNA origami and incubated in the thermocycler (Bio-Rad) at 37 °C for 1 h, followed by keeping the system under room temperature overnight.

#### Step 4: Removal of Excess Conjugate

This was carried out also by using Amicon 100 K filter tubes (Millipore). The removal process included 6 times washings with the folding buffer at 5000*g* for 2 min. The final concentrations of peptide-patterned DNA origami were measured at UV–vis A260 on Nanodrop (Thermo Scientific).

### Agarose Gels Electrophoresis

2% agarose (Sigma-Aldrich) gels were cast in 0.5× TBE buffer (VWR) supplemented with 10 mM MgCl_2_ and 0.5 mg/mL ethidium bromide (Sigma-Aldrich). For all samples, gels were run in 0.5× TBE buffer supplemented with 10 mM MgCl_2_ at 90 V for 3 h on ice. Gels were imaged under a GE LAS 4000 imager.

### Gel-Based Peptide Quantification of DNA Origami

#### Step 1: DNA Origami Digestion

Under 37 °C, 10 μL of 20 nM DNA origami structures (with different numbers of peptide attachment) in PBS were incubated with 0.1 U/μL DNase I (Invitrogen) and 2 mM MgCl_2_ for 25 min. The goal of this process was to completely degrade the DNA origami template, releasing peptides from the structure.

#### Step 2: Gel Electrophoresis and Peptide Staining

The samples were then run on 4–20% gradient PAGE gel (Bio-Rad) with 1 L buffer containing 3 g of Tris base (VWR), 14.4 g of glycine (VWR), and 1 g of SDS (Sigma-Aldrich). After the run, the gel was stained by the method of silver staining.

#### Step 3: Peptide Band Intensity Quantification

In ImageJ, the band areas (same size for all bands on one gel) were selected, and the mean pixel intensities of the areas were used to compare the peptide amount.

#### Step 4: Peptide Occupancy Rate Calculation

We calculated the peptide amount of each sample on the basis of their band intensity on the polyacrylamide gels: peptide amount of each sample = (known peptide amount of peptide only sample × band intensity of each sample)/(band intensity of peptide only sample). We then calculated the protrusion site occupation (by peptide) percentage with the following equation: occupation percentage = [peptide of each sample/(DNA original amount of in each sample × number of protrusion sites)] × 100%.

### Atomic Force Microscopy

Samples of μL each, with the concentration of 1.5 nM in imaging buffer [10 mM MgCl_2_ (Sigma-Aldrich), 5 mM TRIS (VWR), and 1 mM EDTA (VWR)] were dropped to freshly cleaved mica for 30 s incubation. A 4 μL aliquot of NiSO_4_ (5 mM, VWR) was added for a further 5 min incubation. We then washed the sample micasurface by 1 mL of imaging buffer. We used a cantilever AC40 (Bruker) with a nominal spring constant of 0.09 N/m to carry out the AFM (JPK instruments Nanowizard 3 ultra) imaging.

### DNA-PAINT

#### Step 1: Sample Preparation for DNA-PAINT

Glass microscope slides (VWR) and coverslips (1.5H, VWR) were cleaned with acetone and isopropanol before drying. Double-sided Scotch tape was placed onto the slides in two parallel stripes approximately 0.8 cm apart, and the clean coverslips were placed on it to create flow chambers. The channel was flushed with 1 mg/mL biotinylated-BSA (Sigma-Aldrich) in buffer A (10 mM Tris-HCl, 100 mM NaCl, and 0.05% Tween-20, pH 7.5) and incubated for 2 min. The channel was then washed with buffer A. The channel was then flushed with 0.5 mg/mL streptavidin (Thermo Scientific) in buffer A and incubated for 2 min. The channel was washed then with buffer A. After this 80 nm of AuNP solution (Sigma-Aldrich), used as fiducial markers, resuspended in buffer A was flushed into the channel and incubated for 2 min followed by a washing step with buffer A. The channel was then washed with buffer B (5 mM Tris-HCl, 10 mM MgCl2, 1 mM EDTA, and 0.05% Tween-20, pH 8) before structures carrying TRAIL peptides with DNA-PAINT docking sites and 6 biotin sites for immobilization resuspended in buffer B were flushed in at 200 pM concentration and incubated for 5 min, followed by washing with buffer B. The channel was then flushed with imaging buffer and sealed with epoxy glue. The imaging buffer used was based on buffer B and contained oxygen scavengers 2.4 mM PCA (Sigma-Aldrich) and 10 nM PCD (Sigma-Aldrich) and 1 mM Trolox (Sigma-Aldrich) along with Atto-550-labeled imager strands (IDT) at the concentration of 10 nM.

The samples were imaged with a microscope with a Nikon Eclipse Ti-E microscope frame with the Perfect Focus system (Nikon Instruments) and an objective-type TIRF configuration using an iLAS2 circular TIRF module (Gataca Systems). For magnification, a 1.49 NA CFI Plan Apo TIRF 100× oil immersion objective (Nikon Instruments) was used with a 1.5× auxiliary Optovar magnification resulting in a final pixel size of 87 nm. For illumination, an OBIS 561 nm LS 150 mW laser (Coherent) was used with custom iLas input beam expansion optics (Cairn) optimized for reduced field super-resolution imaging. Before the objective, the laser beam was passed through a filter cube (89901, Chroma Technology) containing an excitation quadband filter (ZET405/488/561/640x, Chroma Technology), a quadband dichroic (ZET405/488/561/640bs, Chroma Technology), and a quadband emission filter (ZET405/488/561/640m, Chroma Technology). The collected light was spectrally filtered with an additional emission filter (ET595/50m, Chroma Technology) before entering the iXon Ultra 888 EMCCD camera (Andor) used for recording. The Micromanager software was used for acquiring 12000 frames of long time lapses of samples using frame-transfer mode of the camera, 300 ms exposure time, 10 MHz readout rate, and no EM gain.

#### Step 2: Preprocessing of DNA-PAINT Data

Localization coordinates in the collected time lapses were calculated using the Picasso Localize program from the Picasso software package using 2000 for the minimum net gradient for localization identification and the MLE algorithm with 0.001 and 1000 set as convergence criterion and maximum number of allowed iterations, respectively. The localizations were then drift-corrected using the RCC algorithm with 200 frame fragment size in the Picasso Render program and localizations belonging to the gold nanoparticles used as fiducial markers were selected and exported using the “export selected localizations” feature of the program for later filtering. Following this the low precision localization (localization precision > 0.03 camera pixels), asymmetric localizations (localization ellipticity < 0.1) and multilocalizations (photon count > mean photon count + 2 STD) were removed along localization belonging to the gold nanoparticles using a custom Python script. Finally, the localizations belonging to single structures were selected using the “pick similar” feature of the Picasso Render software (circular pick regions, 1.5 camera pixel diameter, pick similar range of 2.0 std) starting from 30 manually picked structures and the localization were undrifted a second time using the software’s “undrift from picked” feature.

### Processing of DNA-PAINT Images of Peptide-Patterned DNA Origamis

#### Detection of DNA Origami Structure in Localization Data

DNA origami structures were detected using a custom Python script from the cleaned localization data. The localizations were rendered into a low-resolution image (20× oversampling), and clusters of localizations were detected using contour detection and sorted into origami ROIs centered around the detected clusters. ROIs containing noise due to unspecific binding of imagers were removed on the basis of the cluster size and temporal span of localization within the ROI.

#### Quantification of TRAIL Peptides in Structure ROIs

TRAIL proteins were detected using a custom Python script in the localizations sorted into origami ROIs. Localizations in each origami ROI were rendered into high-resolution images (60×, 60×, 100×, 120×, 120×, and 150× oversampling for the 37, 28, 19, 16, 9, and 5 nm origami structures); the intensity of images was normalized and a Gaussian smoothing filter was applied to them. Local maxima in these images were detected, and maxima closer than ∼0.5× the designed site distance were merged. To only keep maxima belonging to a single origami structure, a euclidian distance-based clustering was applied to the maxima and only the most central clusters with the highest number of maxima were kept. The coordinates of these maxima were then exported as detected positions of TRAIL peptides. For quantification, the TRAIL peptide per origami distribution was calculated from counting the detected TRAIL peptides in each origami ROI, and for the mean nearest neighbor distance distribution the distance to the closest neighbor was calculated for each TRAIL position detected in an origami ROI and the mean was calculated from that for each origami ROI in each data set.

### Cell Culture

Cell lines were purchased from ATCC. All of them were cultured in Dulbecco’s modified Eagle’s medium (DMEM, Sigma-Aldrich) with 20% heat-inactivated FBS (Gibco) and 100 U/mL penicillin-streptomycin (Gibco) in a humidified environment containing 5% CO_2_ at 37 °C. To establish GFP-DR5-expressing cell lines, we used Lipofectamine 3000 reagent (Thermo Fisher Scientific) to transfect GFP-tagged human tumor necrosis factor receptor superfamily 10b (GFP-TNFRSF10B/GFP-DR5) plasmid (OriGene) into the cell lines. The GFP sequence is tagged to the C terminus of the sequence of DR5, and thus it locates inside the cytoplasm. For each well of a 6-well plate, cells were cultured to be 70–90% confluent for transfection. Then 2.5 μg of GFP-TNFRSF10B plasmid was mixed with 3.75 μL of Lipofectamine 3000 reagent and 5 μL of P3000 reagent (reagent in the kit) for 15 min incubation. Then we added the DNA–lipid complex to each cell well for 2 days. The transfected cells were then used immediately for the following treatment experiments.

### Confocal Data Collection and Image Analysis

At 24 h prior to the transfection of GFP-DR5 plasmid, 1 × 10^6^ cells per well were cultured on a coverslip in each well of a 6-well plate. After 2 days, cells were washed with fresh DMEM medium containing 20% heat-inactivated FBS and 100 U/mL penicillin-streptomycin. Then the cells were treated with peptide-patterned DNA origami structures (Cy5-free or Cy5-labeled), at certain concentrations and for certain periods (as indicated in corresponding figures or the caption of figures). Finally, cells were fixed in 4% paraformaldehyde for 15 min, washed, and stained by Fluoroshield Mounting Medium with DAPI (Abcam). Cells were imaged on LSM710 (Zeiss). For GFP-DR5 cluster quantification in ImageJ, images were converted into 8-bit images and filtered for the next processing. The absolute scale in micrometers per pixel of the image was set. The clusters were then outlined, counted, and analyzed in ImageJ using the Analyze Particles plug-in. The GFP-DR5 cluster size threshold was 0.500 μm^2^. For colocalization analysis between Cy5-labeled DNA origami and GFP-DR5 clusters, the analysis was performed with Colocalization Analysis plug-in in ImageJ.

### Flow Cytometry

In a 6-well plate, 1 × 10^7^ cells per well were cultured for 24 h prior to treatments. Cells were then treated with 2 nM peptide-patterned DNA origami (equals to 12 nM peptide) for 24 h. All cells (including dead and detached cells that were present in the medium) were collected (centrifuge at 3000 rpm for 5 min) and washed with cold PBS for 3 times. Cells were resuspended and then stained with annexin V-FITC and PI, sequentially, according to the commercial protocol of the Dead Cell Apoptosis Kit with Annexin V-FITC and PI (Thermo Fisher Scientific).

### Cleaved Caspase-8 Detection

We used the method of cell-based ELISA with use of a Human Cleaved Caspase-8 (Asp391) Immunoassay kit (R&D Systems).

#### Step 1: Culture, Treat, Fix, and Block Cells

In 100 μL of DMEM medium per well, 1.5 × 10^4^ cells were seeded in a 96-well black polystyrene microplate with clear bottom (Corning) and cultured overnight. Cells were then treated with 2 nM peptide-patterned DNA origami (equals 12 nM peptide) for 4 h. Cells were then fixed by replacing the medium with 100 μL of 4% formaldehyde in PBS, for 20 min under room temperature. After the fixation, cells were washed by the Wash Buffer, kept in 100 μL of the Quenching Buffer for 20 min, again washed by the Wash Buffer, and kept in 100 μL of Blocking Buffer for 1 h.

#### Step 2: Incubation of Primary and Secondary Antibodies

After removing the Blocking Buffer and washing the cells with Wash Buffer, 100 μL of the primary antibody mixture containing rabbit anticleaved caspase-8 (Asp391) and mouse antitotal GAPDH was added for 16 h incubation under 4 °C. The primary antibodies were removed, and the cells were washed by Wash Buffer. Then 100 μL of the secondary antibody mixture containing HRP-conjugated antirabbit IgG and AP-conjugated antimouse IgG was added for 2 h incubation under room temperature to bind targeting primary antibodies.

#### Step 3: Fluorogenic Detection

The secondary antibodies were removed, and the cells were washed with Wash Buffer. A 75 μL aliquot of substrate of HRP was added for 30 min incubation at room temperature, followed by adding 75 μL of substrate of AP for an additional 30 min incubation. The signals were then read under a multimode microplate reader (Varioskan LUX): with excitation at 540 nm and emission at 600 nm for Cleaved Caspase-8 (Asp391) detection; with excitation at 360 nm and emission at 450 nm for total GAPDH detection.

### Cell Viability Assay

We used the method of ATP-based luminescent cell viability assay with the kit named CellTiter-Glo Luminescent Cell Viability Assays (Promega). In 100 μL of DMEM medium per well 5 ×10^4^ cells were seeded in a 96-well opaque white polystyrene microplate (Corning) and cultured for 24 h. Cells were treated with various concentrations of peptide-patterned DNA origami structures (as indicated in corresponding figures or the caption of figures). After 48 h incubation, the plate was taken out from the cell incubator and equilibrated at room temperature for 30 min. A 100 μL aliquot of CellTiter-Glo reagent (Promega) was added to each well, followed by mixing for 2 min on an orbital shaker to induce cell lysis. The plate was then incubated at room temperature for 10 min to stabilize the luminescent signal. Finally, the luminescence was recorded on a multimode microplate reader (Varioskan LUX). The results of control wells containing medium without cells were used as the background luminescence. % viable cells = (luminescence_sample_ – luminescence_background_)/(luminescence_PBS_ – luminescence_background_) × 100.

### Statistical Analysis

All experiments were performed in multiple distinct replicates, as indicated in the text and figure legends. Statistical analysis was performed using *R*. Cluster counting data in the microscopy images were analyzed using Kruskal–Wallis one-way analysis of variance and subsequent Mann–Whitney U tests for further analysis between conditions. Other data were analyzed using two-tailed Student’s *t* tests for 2 groups and one-way ANOVA followed by Tukey post-tests for multiple groups. Results are expressed as mean ± SD unless otherwise indicated. For each box-and-whisker plot, the center line is the median and whiskers represent the minimum and maximum values.

## Supporting Information Available

Structural information and design (Figures S1, S2, and S4); agarose gel electrophoresis (Figures S3, S12, and S13), peptide–DNA conjugation scheme (Figure S5); conjugates gel electrophoresis (Figure S6); clusters interpretation (Figure S7); pattern and template designs (Figures S8–S10); nucleotide-to-nucleotide distance measurement (Figure S11); DNA-PAINT data preprocessing (Figure S14); localization properties and their respective filtering cutoffs (Figure S15); origami ROI coordinates and peptides (Figures S16 and S17); DNA-PAINT images (Figures S18–S23); calculated protruding DNA site occupancy (Figure S24); interpeptide distance distribution (Figure S25); gel-based peptide quantification (Figure S26); DR5 clusters and cleaved caspase-8 induced by L6 (Figures S27 and S28); peptide IC_50_ (Table S1) (PDF)

## Notes

An earlier version of this work was uploaded to a preprint server: WangY.; BaarsI.; FördösF.; HögbergB.Clustering of death receptor for apoptosis using nanoscale patterns of peptides. bioRxiv Preprint (Bioengineering), 2020. https://www.biorxiv.org/content/10.1101/2020.11.16.359729v1 (accessed 2021-05-17)10.1021/acsnano.0c10104PMC822348934019379
