# Supplementary material for: Clustering of Death Receptor for Apoptosis Using Nanoscale Patterns of Peptides
Source: ACS Nano. 2021 May 21;15(6):9614–26. doi: 10.1021/acsnano.0c10104 (PMC8223489; doi:10.1021/acsnano.0c10104)
Supplement: Supplementary file 1 — nn0c10104_si_001.pdf [file nn0c10104_si_001.pdf]

Supporting Information

Clustering of Death Receptor for Apoptosis Using Nanoscale Patterns of Peptides

Yang Wang, Igor Baars, Ferenc Fördös, Björn Högberg*

Department of Medical Biochemistry and Biophysics, Karolinska Institutet, SE-17177 Stockholm, Sweden

*Correspondence to: bjorn.hogberg@ki.se

Index	Contents
Figure S1	Design of L-structure
Figure S2	Computational 3D structure of L designs
Figure S3	Agarose gel electrophoresis of W and L
Figure S4	Basic structural information of the peptide and TRAIL
Figure S5	Scheme of peptide-DNA conjugation
Figure S6	Conjugates gel electrophoresis followed by silver staining
Figure S7	Current interpretation of apoptosis-driving hexagonal DR5 clusters
Figure S8	Illustration on pattern designs on W
Figure S9	Design of the template for L6
Figure S10	Design of the template for L11
Figure S11	Nucleotide-to-nucleotide distance measurement through oxDNA
Figure S12	Agarose gel electrophoresis of peptide-attached W structures
Figure S13	Agarose gel electrophoresis of peptide-attached L structures
Figure S14	Pre-processing of DNA-PAINT data
Figure S15	Plots of localization properties and their respective cutoffs for filtering
Figure S16	Detection of DNA origami ROI coordinates from DNA-PAINT data
Figure S17	Detection of peptides in origami ROIs extracted from DNA-PAINT data
Figure S18	DNA-PAINT images of W37 structures
Figure S19	DNA-PAINT images of W26 structures
Figure S20	DNA-PAINT images of W19 structures
Figure S21	DNA-PAINT images of W16 structures
Figure S22	DNA-PAINT images of W9 structures
Figure S23	DNA-PAINT images of W6 structures
Figure S24	Protruding DNA site occupancy calculated from DNA-PAINT
Figure S25	Inter-peptide distance distribution from DNA-PAINT
Figure S26	Gel-based peptide quantification of L6
Figure S27	DR5 clusters induced by L6 attached with different number of peptides
Figure S28	Cleaved caspase-8 induced by L6 attached with different number of peptides
Table S1	The half maximal inhibitory concentration (IC_{50}) of peptide

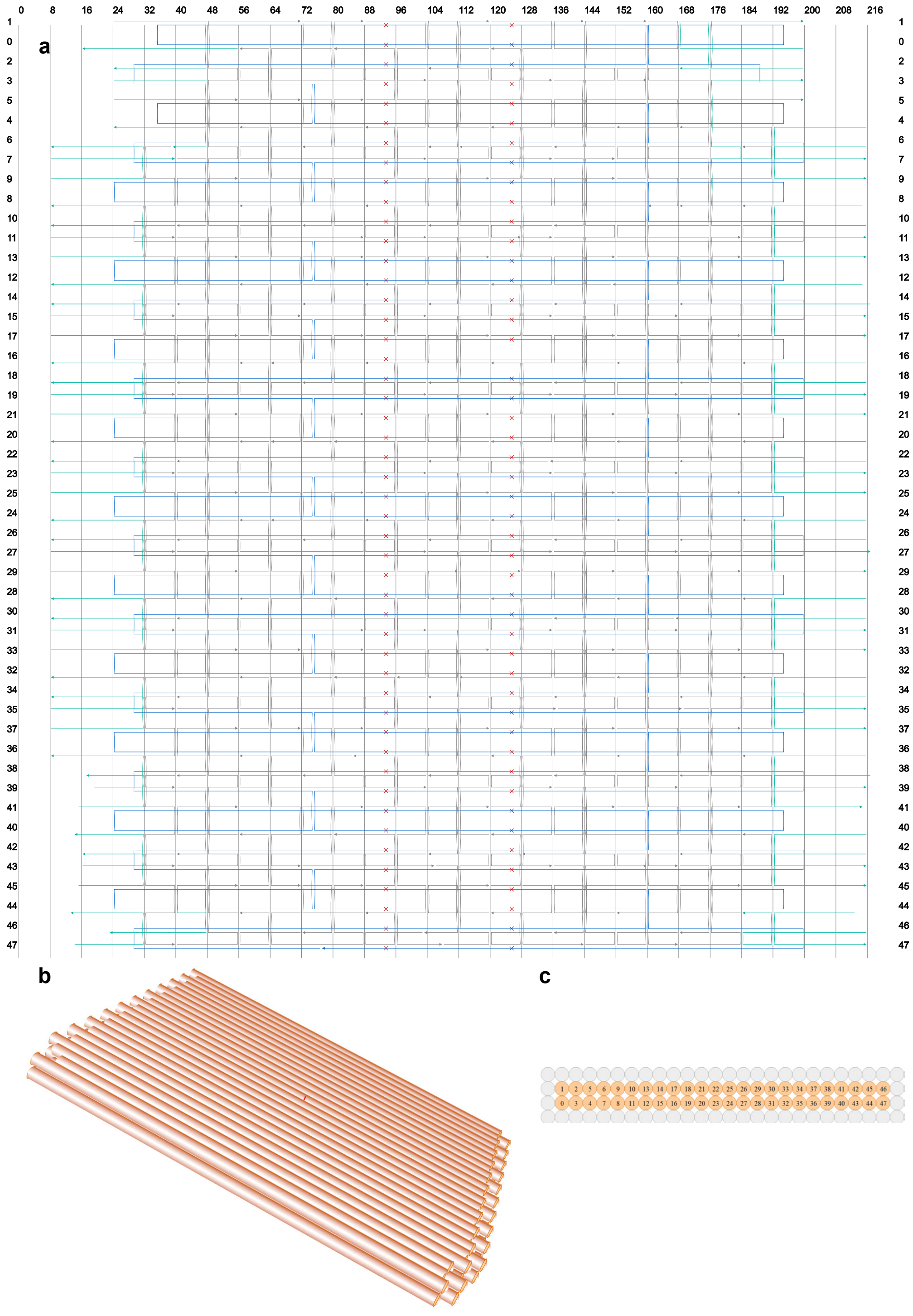

Figure S1. Design of L. (a) the design blueprint of L in cadnanoSQ. The scaffold DNA is in blue, while DNA staples are in gray. The DNA staples colored in cyan have polyA sequences at their non-hybridized parts, which can help reducing aggregation. (b) 3D view of L. (c) the cross-section view of L in cadnanoSQ.

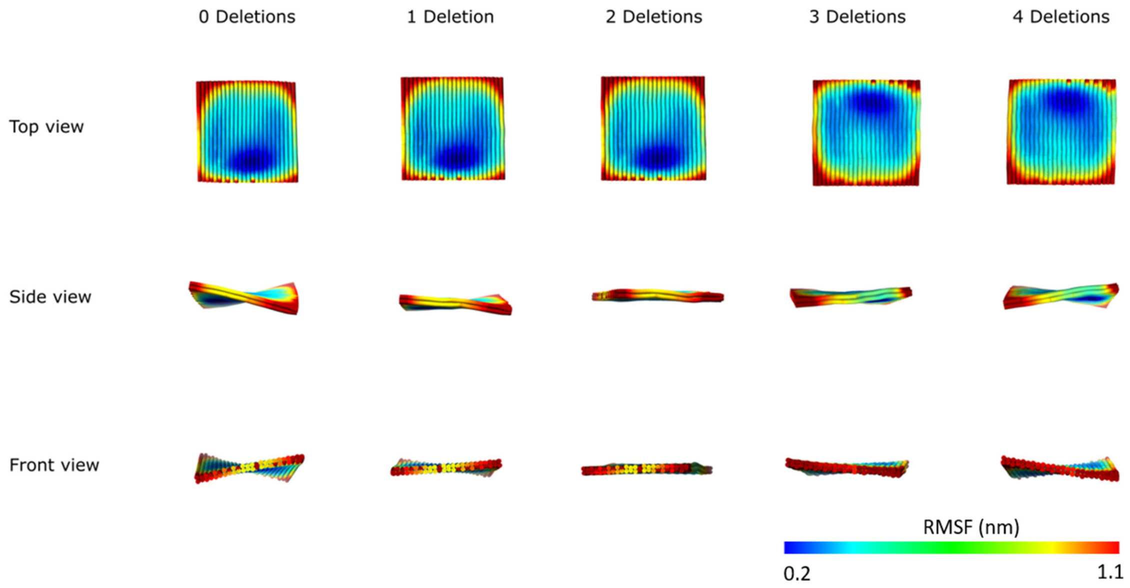

Figure S2. The computational feedback on the 3D structure of different L designs. As shown in Extended Fig.1, there are 2 columns of deleted bases, where the red "×" marks locate, in the scaffold DNA routing. We chose the number of columns for bases deletions by checking the 3D structure prediction in CanDo (https://cando-dna-origami.org/). The template with 2 columns of base deletions (2 Deletions) shows a relatively flat 3D configuration, while other designs (0 Deletions, 1 Deletions, 3 Deletions and 4 Deletions) present twisted configurations. Thus, we used the template having 2 Deletions for next peptide patterning.

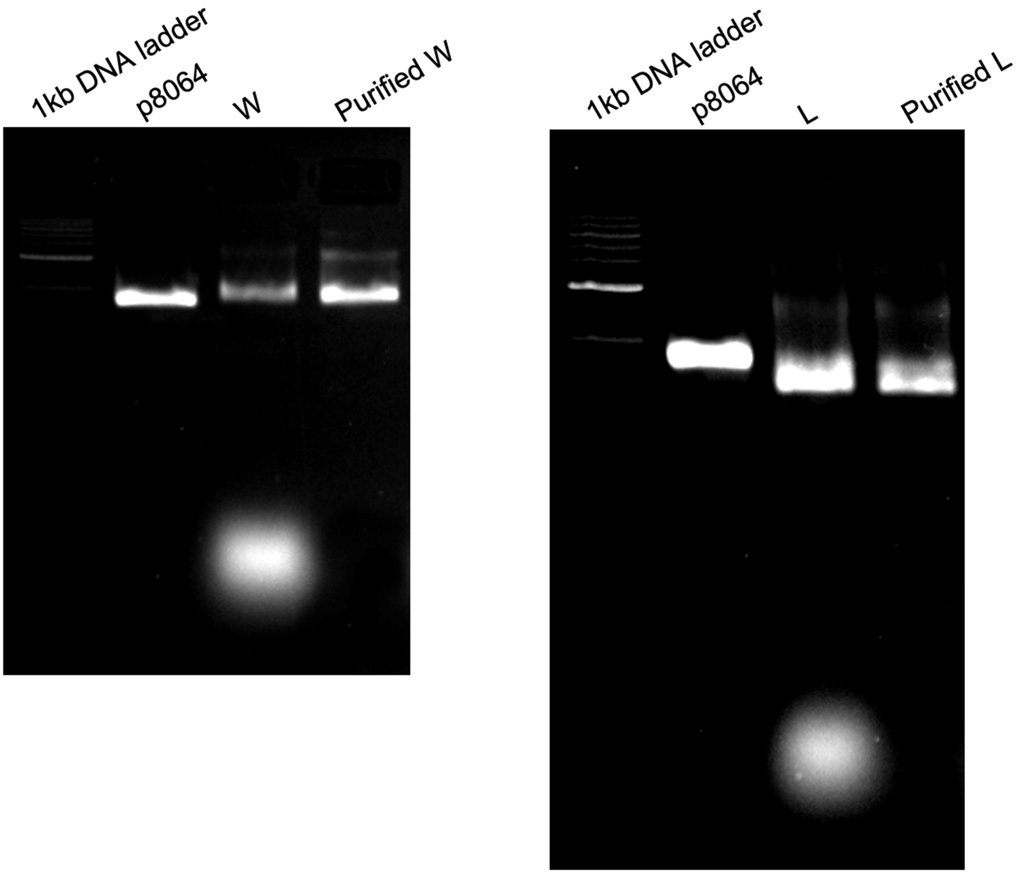

Figure S3. 2% agarose gel electrophoresis of W (Left) and L (Right), before and after purification. The band (the bottom one) stands for the DNA staples, which were completely washed away by the purification process.

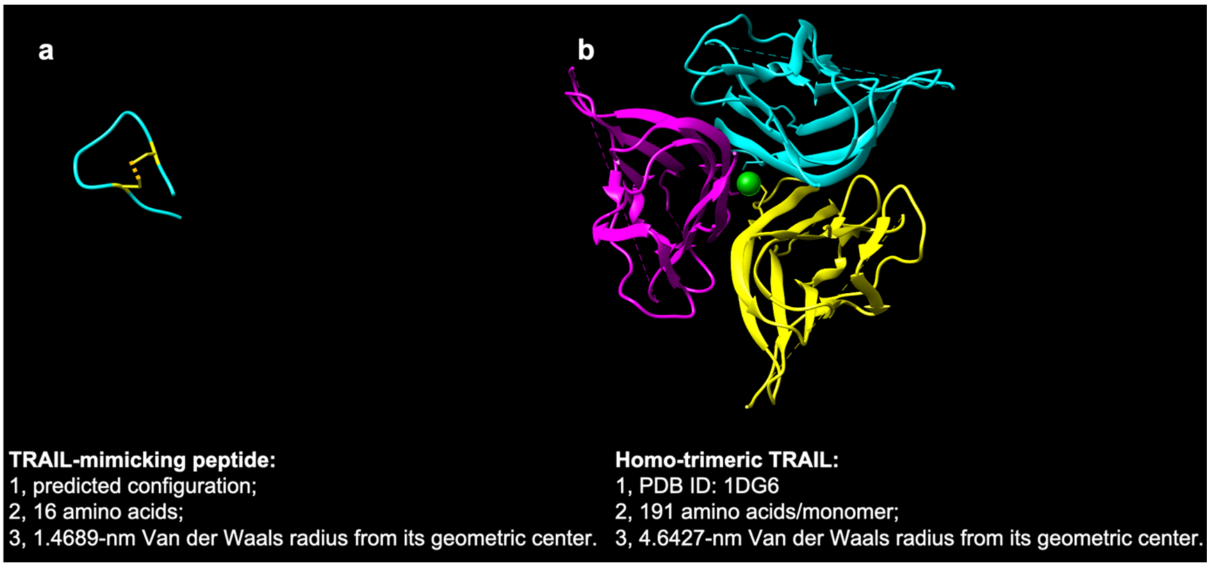

Figure S4. Basic structural information of the peptide and TRAIL trimer. (a) peptide; (b) homo-trimeric TRAIL. To predict the spatial structure of the peptide, its sequence (WDCLDNRIGRRQCVKL) was submitted to PEP-FOLD server (https://bioserv.rpbs.univ-paris-diderot.fr/services/PEP-FOLD/) to compute its PDB coordinates information, which was then visualized and analyzed in Chimera (https://www.cgl.ucsf.edu/chimera/) and YASARA (http://www.yasara.org/). The peptide is cyclized by a disulfide bond between two cysteines. The Human TRAIL trimer structure, with 1DG6 as its PDB ID, was directly sourced from RCSB PDB (https://www.rcsb.org/). Its parameter of Van der Walls radius from its geometric center was also analyzed in YASARA.

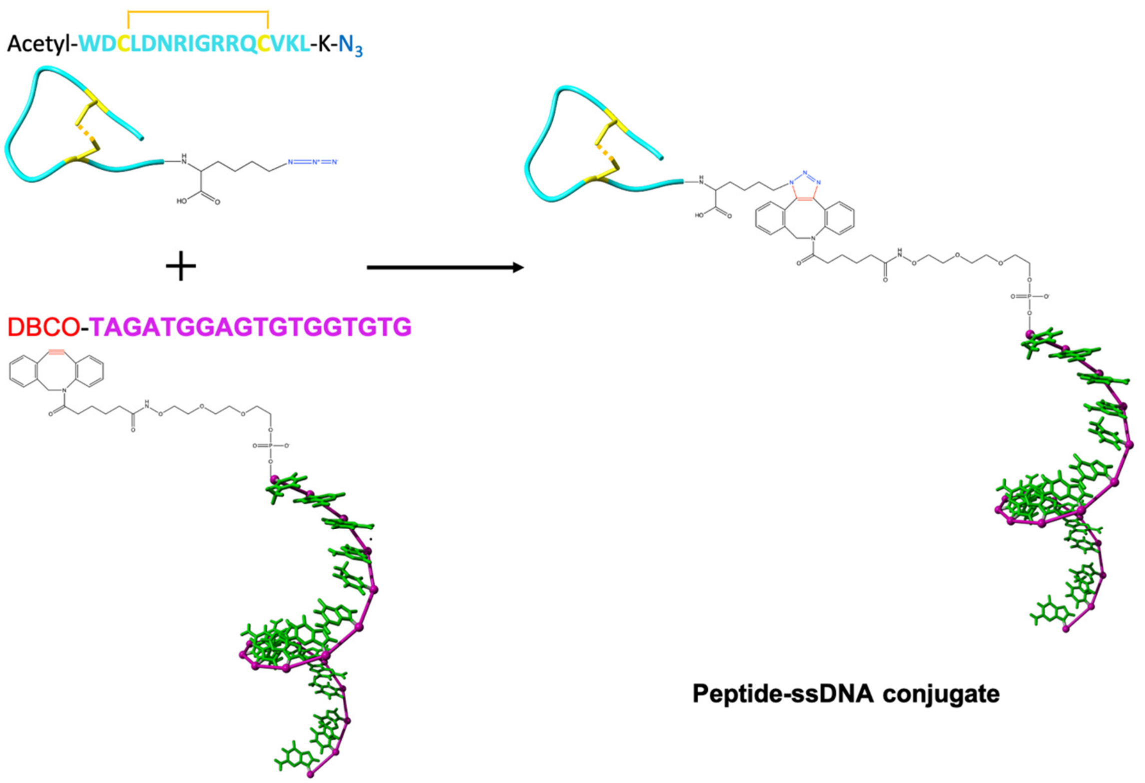

Figure S5. Peptide-DNA conjugation. The peptide is modified with an azide group at its C-terminal; the 18-base long ssDNA is tagged a DBCO at its 5-prime. The reaction is a copper free click chemistry reaction, which has a high reaction efficiency in PBS (pH 7.4), under room temperature.

Lane	1	2	3	4	5	6	7	8
Sample	DNA ladder	DBCO-DNA	Peptide	Conjugate	Conjugate + Cy5-comDNA	Cy5-comDNA	Cy5-comDNA + DBCO-DNA	DNA ladder

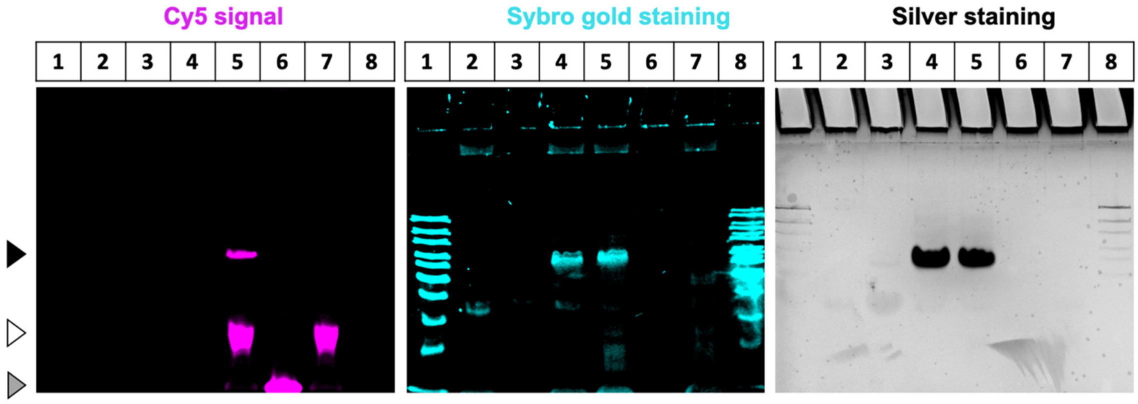

▶ : Conjugate position on the gel
▷ : Formed double-stranded DNA position on the gel
▶ : Cy5-comDNA position on the gel

Figure S6. Validation the peptide-DNA conjugation by gel followed by silver staining. 18% native polyacrylamide gel was used. We used a Cy5-labled complementary DNA (Cy5-comDNA) to hybridize the DNA of the conjugate, thus we can visualize the conjugate under Cy5 channel. All of the samples are indicated as in the figure. The gel was imaged under Cy5 channel; nucleic acids on the gel was stained by SYBR gold; peptides were stained by silver staining.

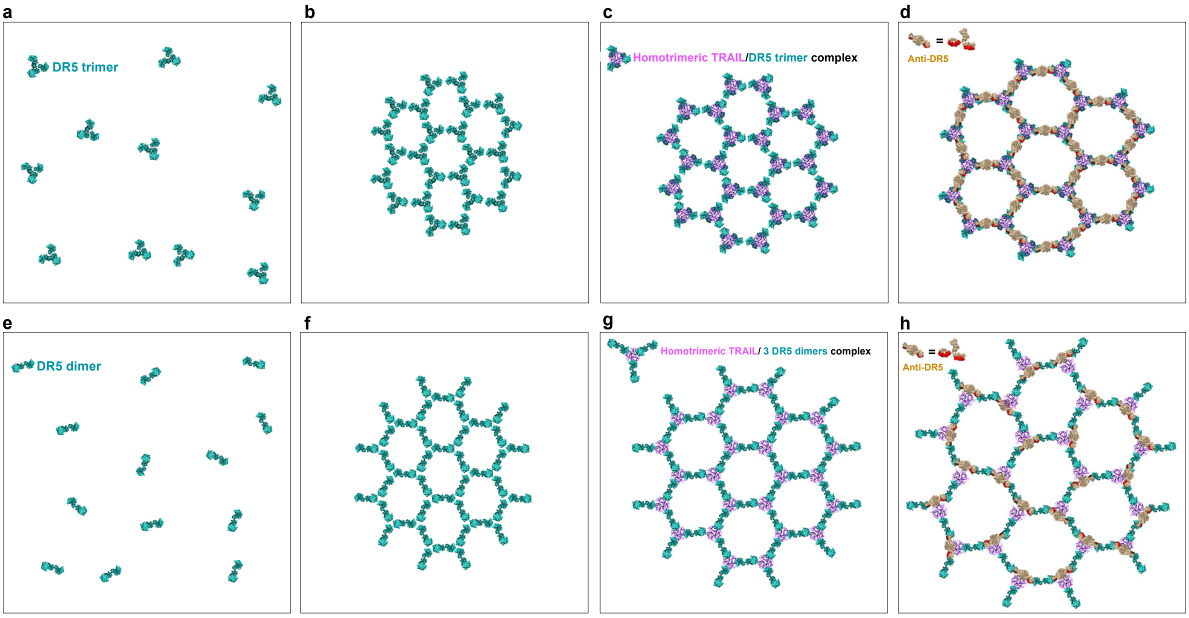

Figure S7. Current interpretation of apoptosis-driving hexagonal DR5 networks (top view from the outside of a cell surface). (a) Surface representations of pre-ligand DR5 trimers. (b) The proposed active hexagonal DR5 network *via* dimerization of DR5 trimers. (c) The proposed active hexagonal network of homo-trimeric TRAIL/DR5 trimer complexes. (d) The hexagonal homo-trimeric TRAIL/DR5 trimer network promoted and maintained by the antibody AMG 655. (e) Surface representations of pre-ligand DR5 dimers. (f) The proposed active hexagonal DR5 network *via* trimerization of DR5 dimers. (g) The proposed active hexagonal network of homo-trimeric TRAIL/DR5 dimer complexes. (h) The hexagonal homo-trimeric TRAIL/DR5 dimer network promoted and maintained by antibody AMG 655.

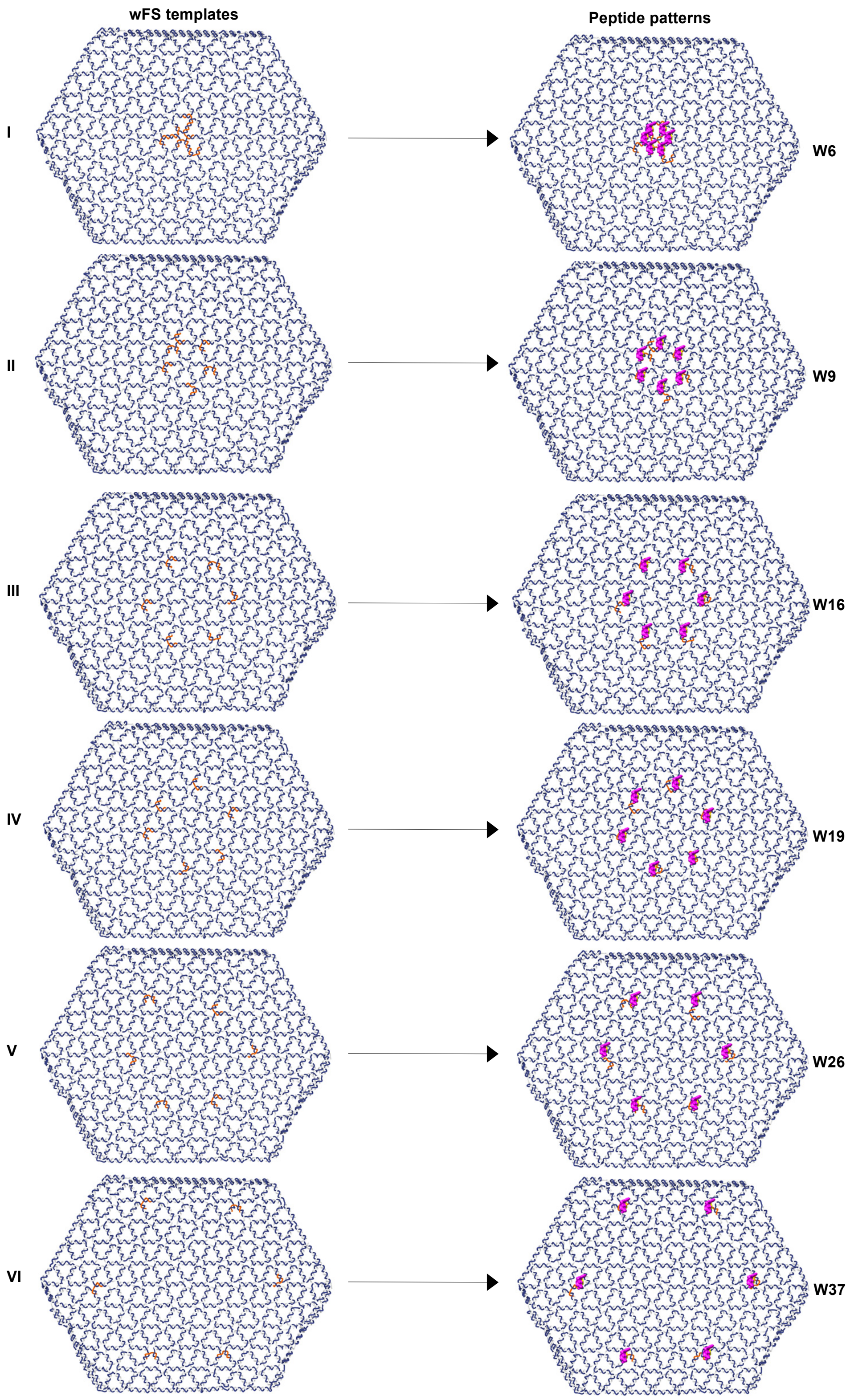

Figure S8. Designs of peptide patterns on W. The sizes of hexagonal patterns are decided by the choices of specific DNA staples on W. These staples were colored in orange, while blue is the scaffold DNA and gray means other DNA staples. These staples in orange contain protruding single-stranded DNA at their 5 primes, thus they can hybridize with the DNA-peptide conjugate.

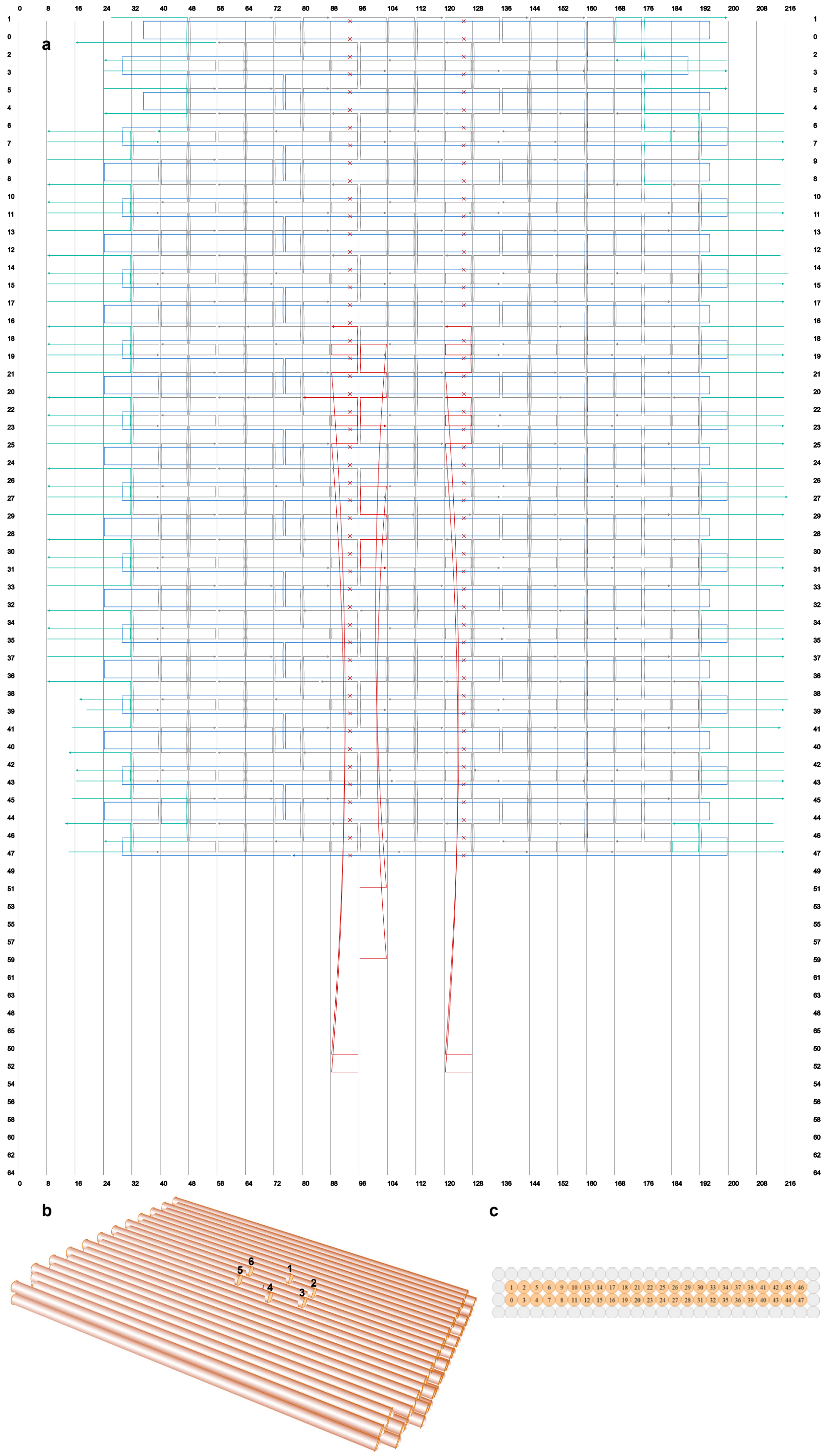

Figure S9. Design of the template for L6. a, the design blueprint of the template in cadnanoSQ. The scaffold DNA is in blue, while DNA staples are in gray. The DNA staples colored in cyan have polyA sequences at their non-hybridized parts, which can help reducing aggregation. The 6 staples having protruding sequences at their 5 primes, for the docking of DNA-peptide conjugate, were colored in red. b, the 3D view of the tempalte, showing that the 6 protruding sites are presenting a hexagonal pattern, and the distance between adjacent sites are around 5 nm. c, the cross-section view of the template in cadnanoSQ.

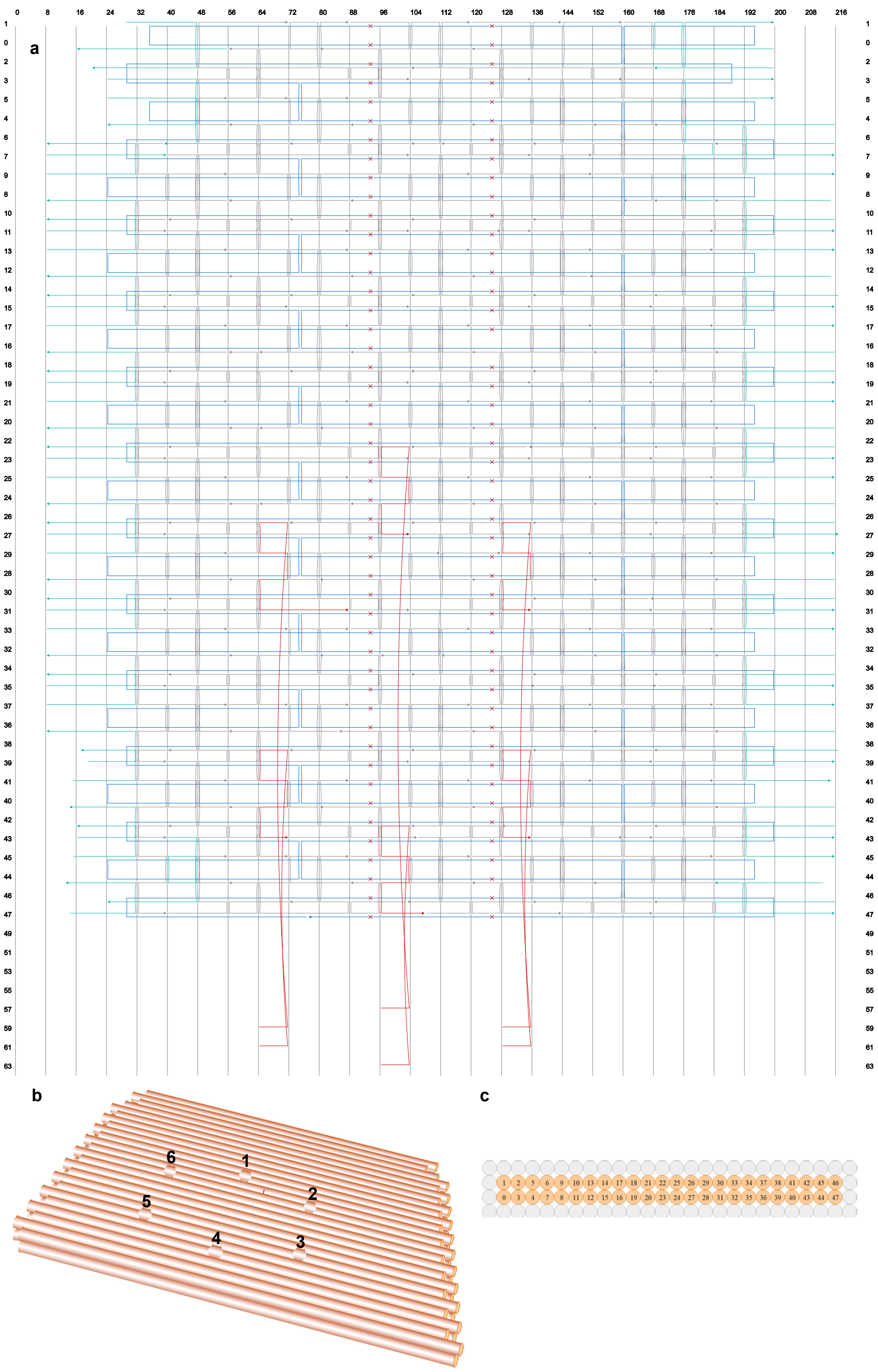

Figure S10. Design of the template for L11. (a) the design blueprint of the template in cadnanoSQ. The scaffold DNA is in blue, while DNA staples are in gray. The DNA staples colored in cyan have polyA sequences at their non-hybridized parts, which can help reducing aggregation. The 6 staples having protruding sequences at their 5 primes, for the docking of DNA-peptide conjugate, were colored in red. (b) the 3D view of the tempalte, showing that the 6 protruding sites are presenting a hexagonal pattern, and the distance between adjacent sites are around 10 nm. (c) the cross-section view of the template in cadnanoSQ.

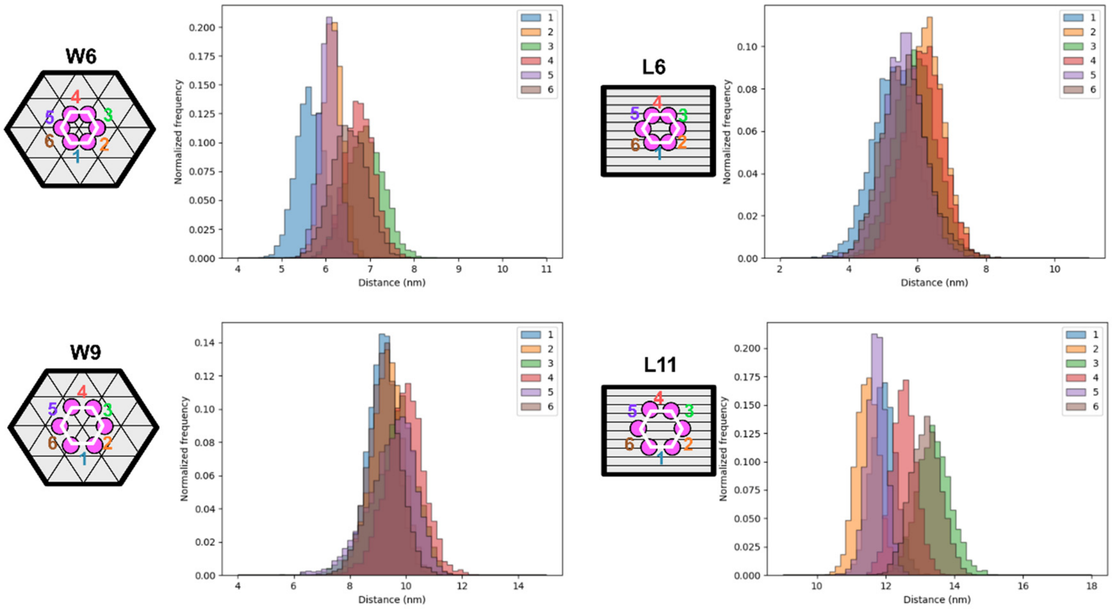

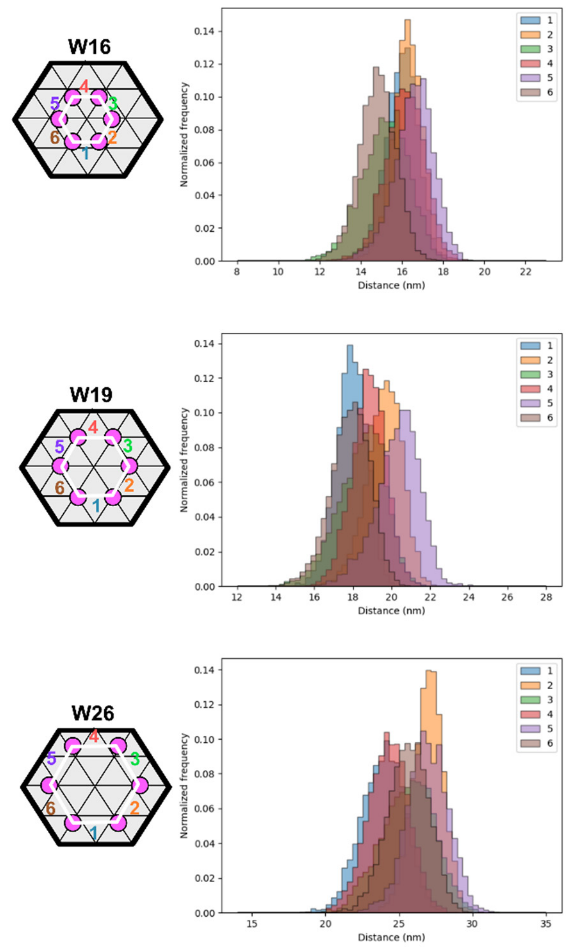

Name	Edge 1	Edge 2	Edge 3	Edge 4	Edge 5	Edge 6	Mean	SD
W6	5.65	6.16	6.89	6.76	6.06	6.52	6.34	0.46
W9	9.15	9.32	9.55	9.94	9.44	9.21	9.43	0.95
W16	15.95	16.27	15.18	16.14	16.56	14.79	15.82	0.68
W19	17.89	19.62	18.41	18.8	20.3	17.83	18.8	0.98
W26	23.88	26.93	25.58	24.25	26.9	25.62	25.53	1.28
L6	5.33	6.22	5.91	5.45	5.58	5.62	5.68	0.32
L11	12.01	11.12	11.22	11.53	12.72	12.16	11.79	0.28

Figure S11. Nucleotide-to-nucleotide distance measurement through oxDNA molecular dynamics simulation. For each hexagonal pattern on DNA origami, the distance distribution (within the oxDNA simulation time frame) of each edge is shown in the distribution histograms.

The mean distance of each edge, the mean edge distance of a hexagonal pattern and corresponding standard deviation (SD) are listed in the table.

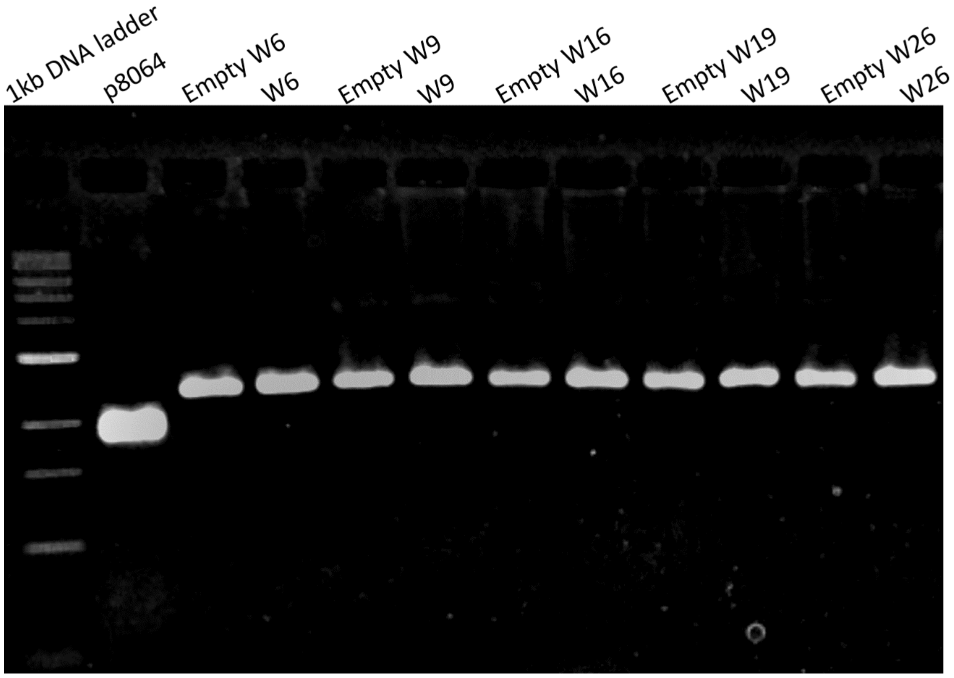

Figure S12. 2% agarose gel electrophoresis of all W (with and without peptide attachment) structures. The sample of each well is indicated as in the figure.

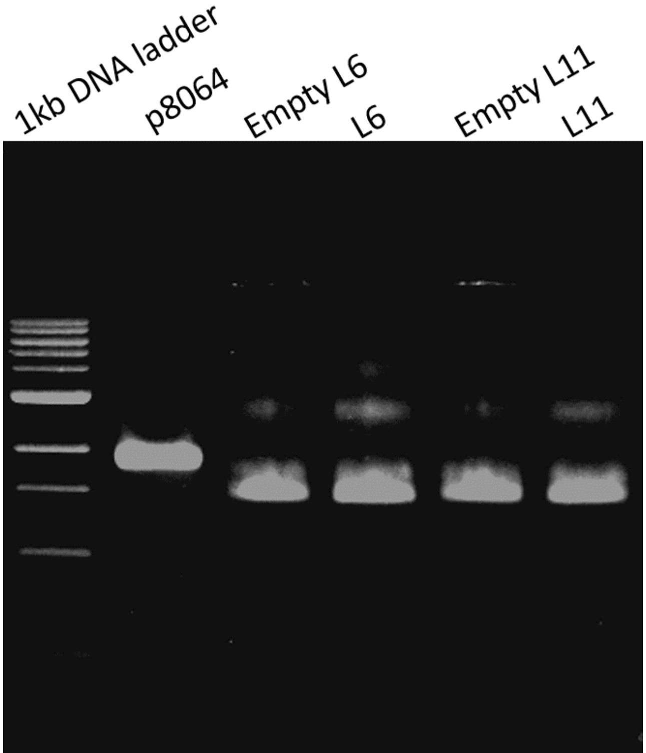

Figure S13. 2% agarose gel electrophoresis of all L (with and without peptide attachment) structures. The sample of each well is indicated as in the figure.

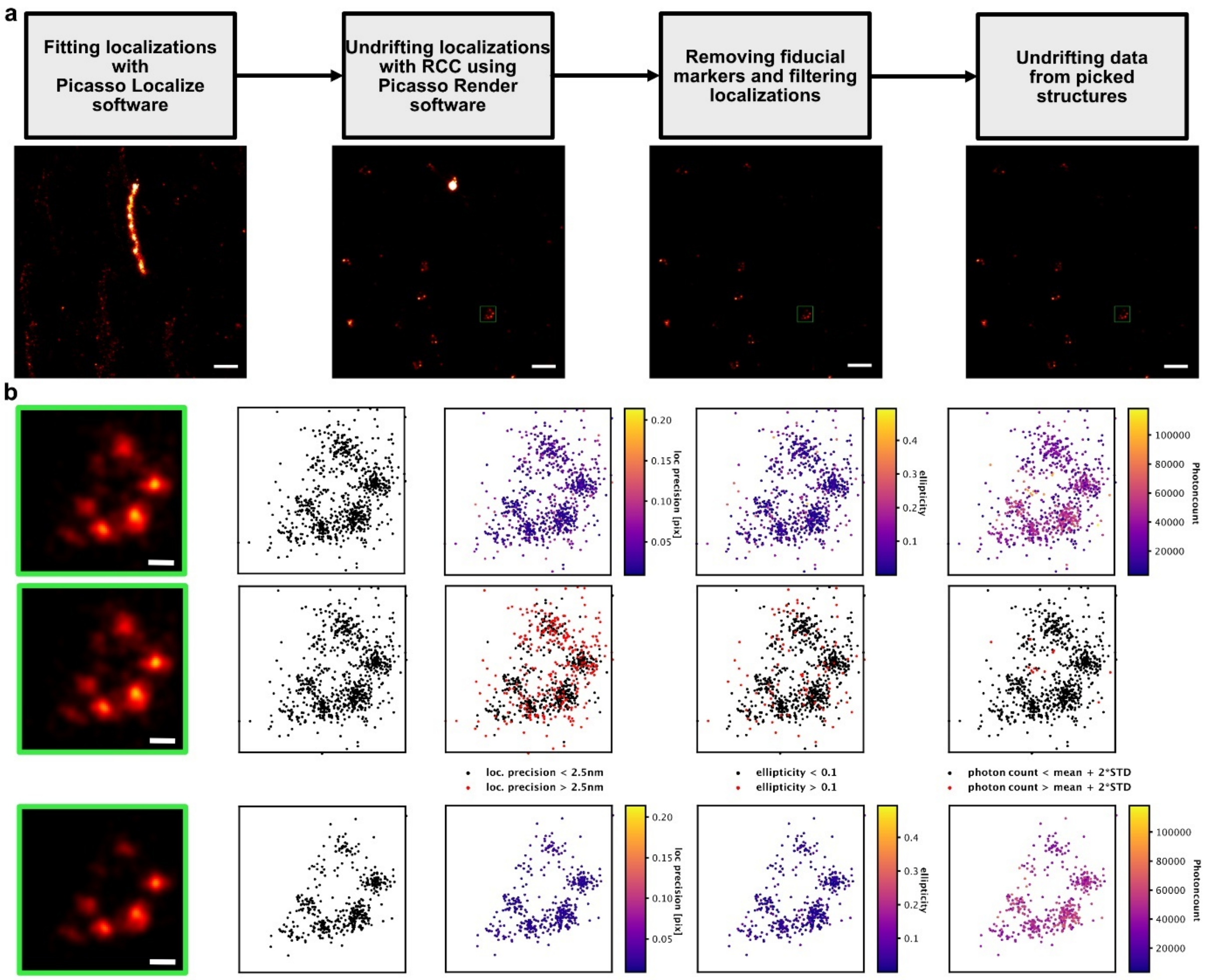

Figure S14. Pre-processing of DNA-PAINT data. (a) Steps of preprocessing the DNA PAINT data with super resolved field of view images of the resulting DNA PAINT localization data of the TRAIL-peptide functionalized wFS37 structures (scale bar=200nm). (b) Plots of localizations belonging to a TRAIL-peptide functionalized wFS37 structure taken from the FOV image (green rectangle) showing localization properties before filtering (upper row), localizations removed by the corresponding filters (red) (middle row) and localization properties after filtering (bottom row) along the super resolution image rendered from the corresponding localizations (scale bar = 20nm).

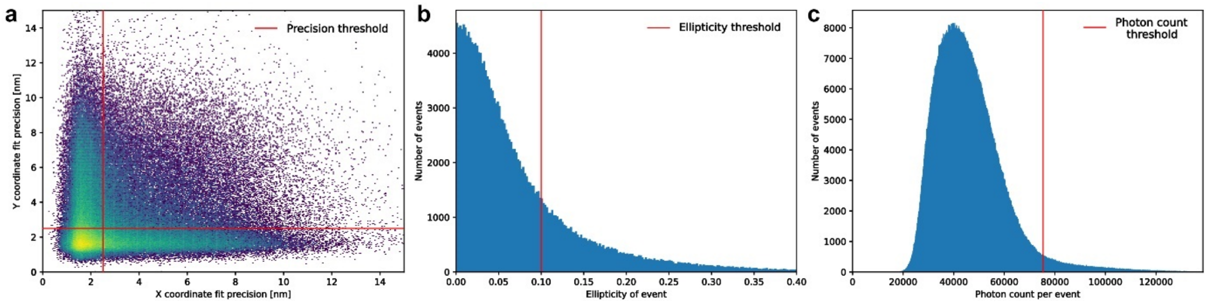

Figure S15. Plots of localization properties and their respective cutoffs used for filtering. (a) Plots of localization fitting precision, (b) ellipticity , (c) and photon counts of the localization data produced by imaging TRAIL-peptide functionalized wFS37 structure with the thresholds applied during preprocessing indicated.

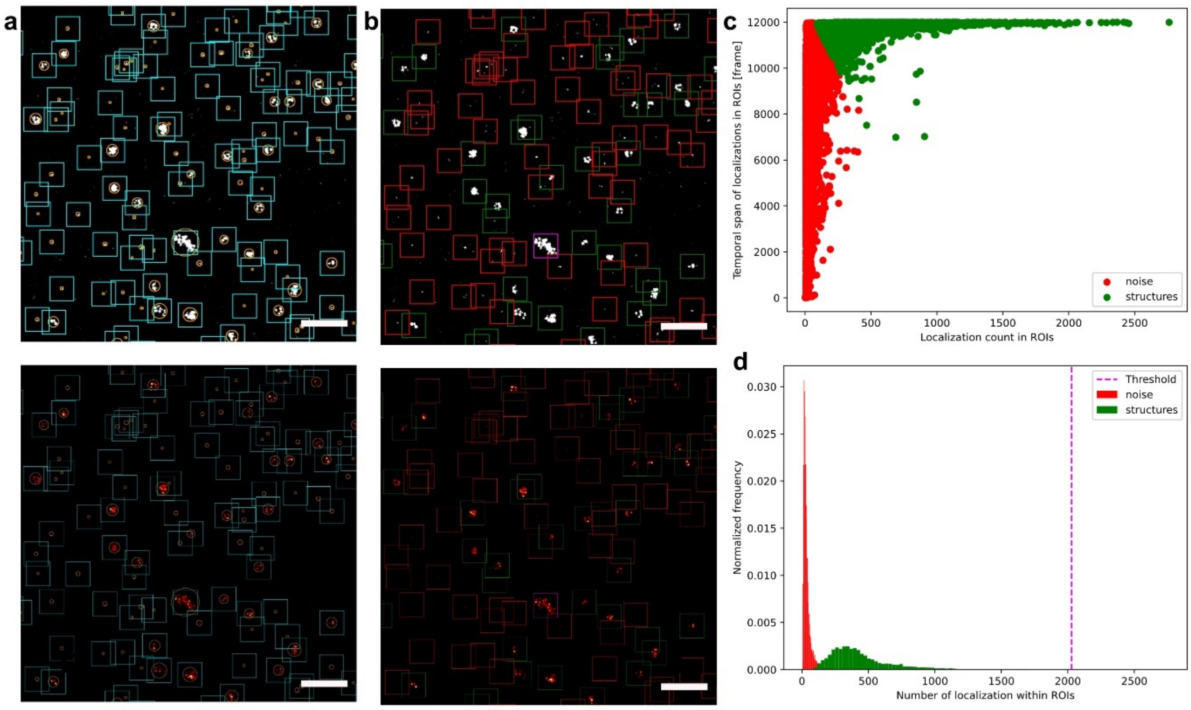

Figure S16. Detection of DNA origami ROI coordinates from DNA-PAINT data (A-D) Steps for DNA origami structure position detection in DNA PAINT data presented on the gray scale (top row) and colormap FOV images (bottom row) of W37 structures (scalebars = 500nm). (a) Possible positions of origamis structures were found by detecting contours (orange circles) in the grayscale low resolution images rendered from the data and uniform origami ROI coordinates (cyan boxes) were calculated using the centroid coordinates of the contours. (b) The origami ROIs containing noise (red boxes) were filtered out based on the number and temporal span of localization in them, ROIs containing aggregates of structures (magenta boxes) were filtered out by removing ROIs containing 10 STD more localization than the mean of non-noise containing ROIs, the remaining ROIs (green boxes) were kept as origami ROIs for further processing. (c) Scatter plot of ROI localization numbers and temporal spans clustered into 2 groups using k-means clustering on two-component PCA transformed data resulting in a cluster of ROIs with shorter temporal span containing noise (red) and ROIs containing signal from structures. (d) Distribution plot of localization numbers of clustered ROIs showing the

localization number threshold (magenta dashed line) above which ROIs containing aggregates of structures were filtered out.

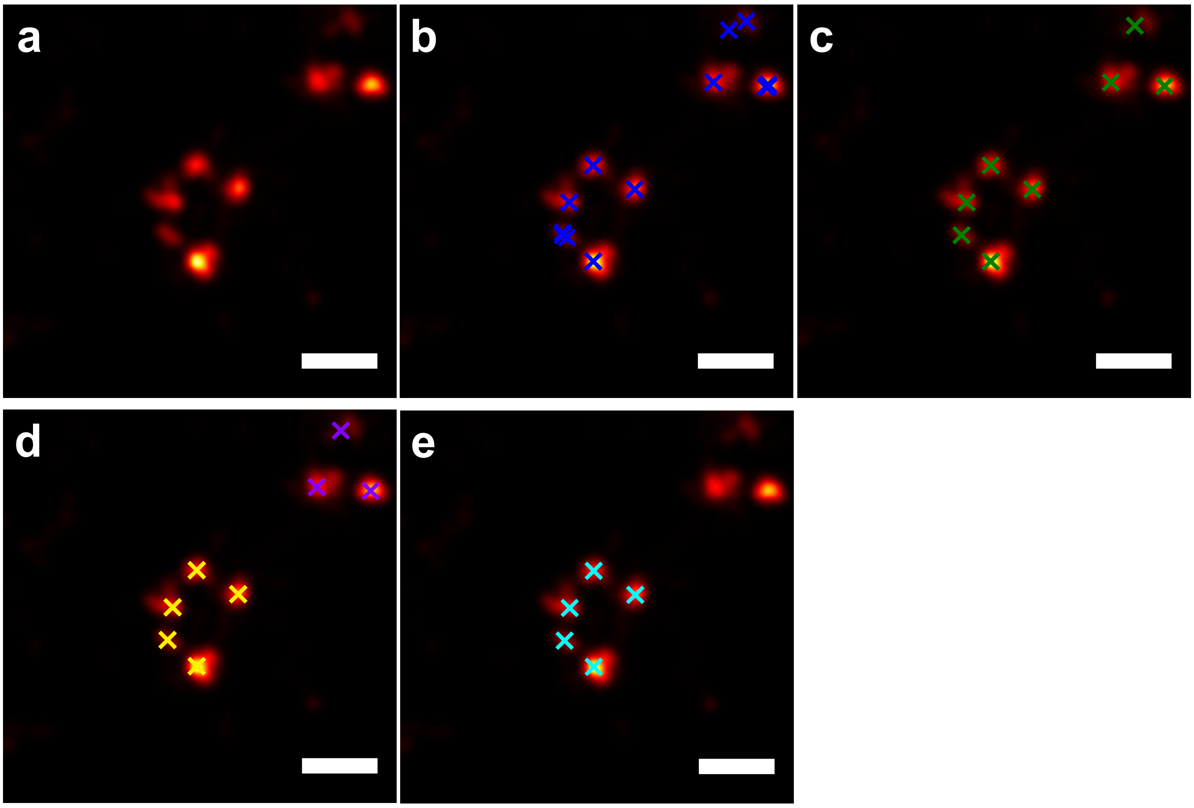

Figure S17. Detection of TRAIL peptides in origami ROIs extracted from DNA-PAINT data. (a) Localizations lying within detected origami ROIs were grouped and rendered. (b) Local maxima (blue crosses) were detected in the rendered image. (c) Maxima lying closer to each other than a distance threshold (0.45 site distance) were merged and an average position for them were calculated yielding the estimated position of TRAIL peptides in the ROI (green crosses). (d) TRAIL peptides belonging to individual origamis were grouped together (purple and yellow crosses) *via* hierarchical clustering with the maximum intra cluster distance set to 2.5 site distance. (e) Finally the cluster with the most number of TRAIL peptides closest to the center of the origami ROI is kept and used for quantification. (Scale bars = 50nm).

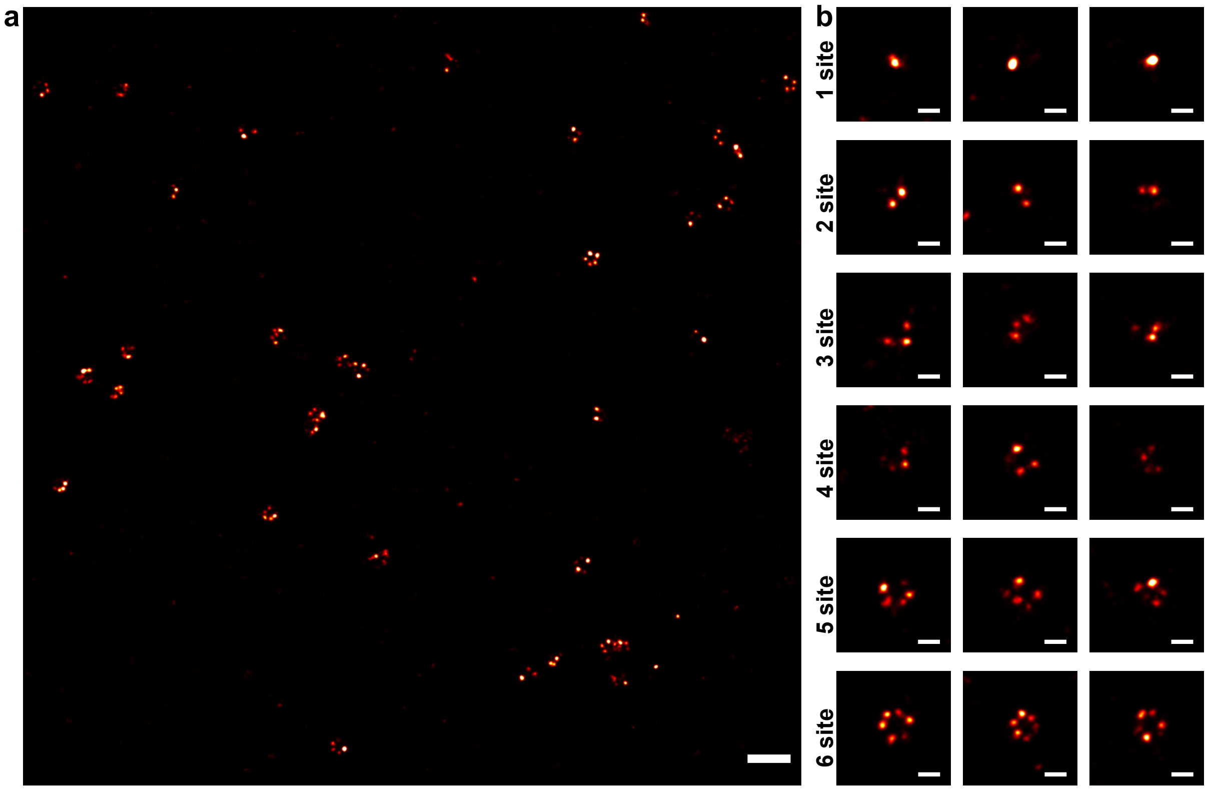

Figure S18. DNA-PAINT images of W37 structures. (a) A field of view DNA PAINT image of the W37 structures (Scale bar = 200nm). (b) Rendered images of origami ROIs containing 1, 2, 3, 4, 5 and 6 detected TRAIL peptides (Scale bar = 50nm).

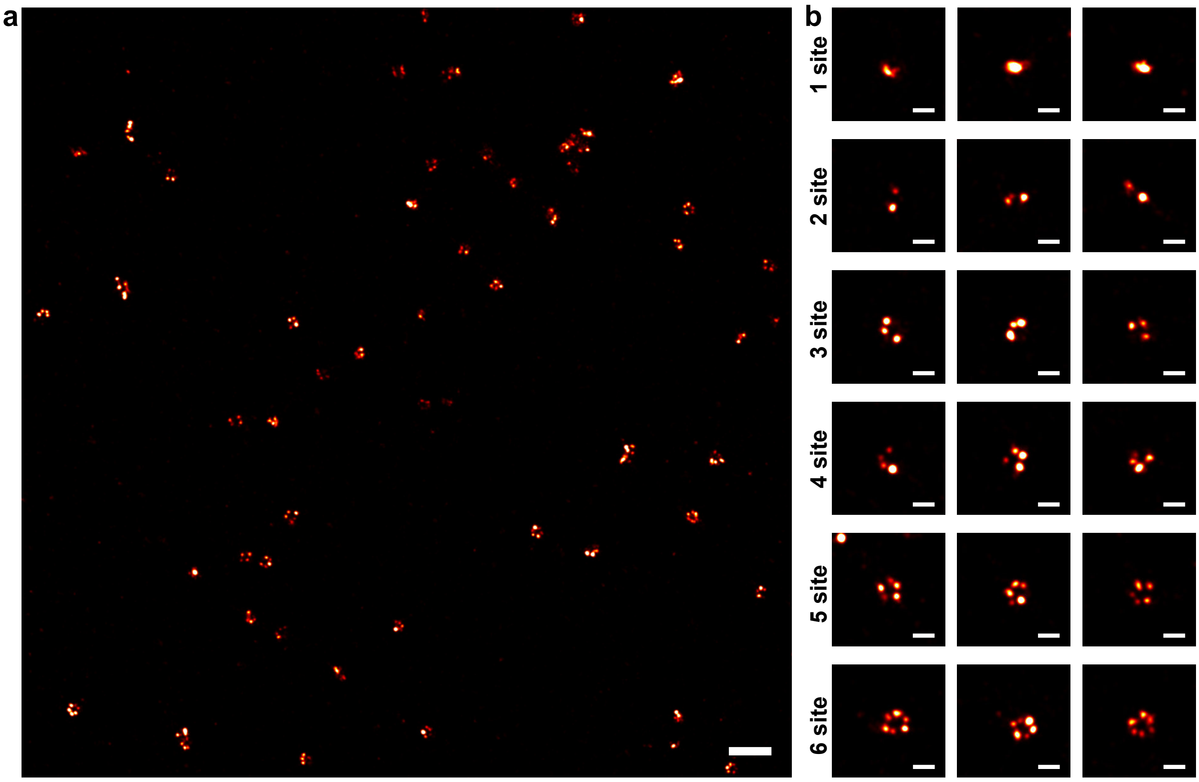

Figure S19. DNA-PAINT images of W28 structures. (a) A field of view DNA PAINT image of the W28 structures (Scale bar = 200nm). (b) Rendered images of origami ROIs containing 1, 2, 3, 4, 5 and 6 detected TRAIL peptides (Scale bar = 50nm).

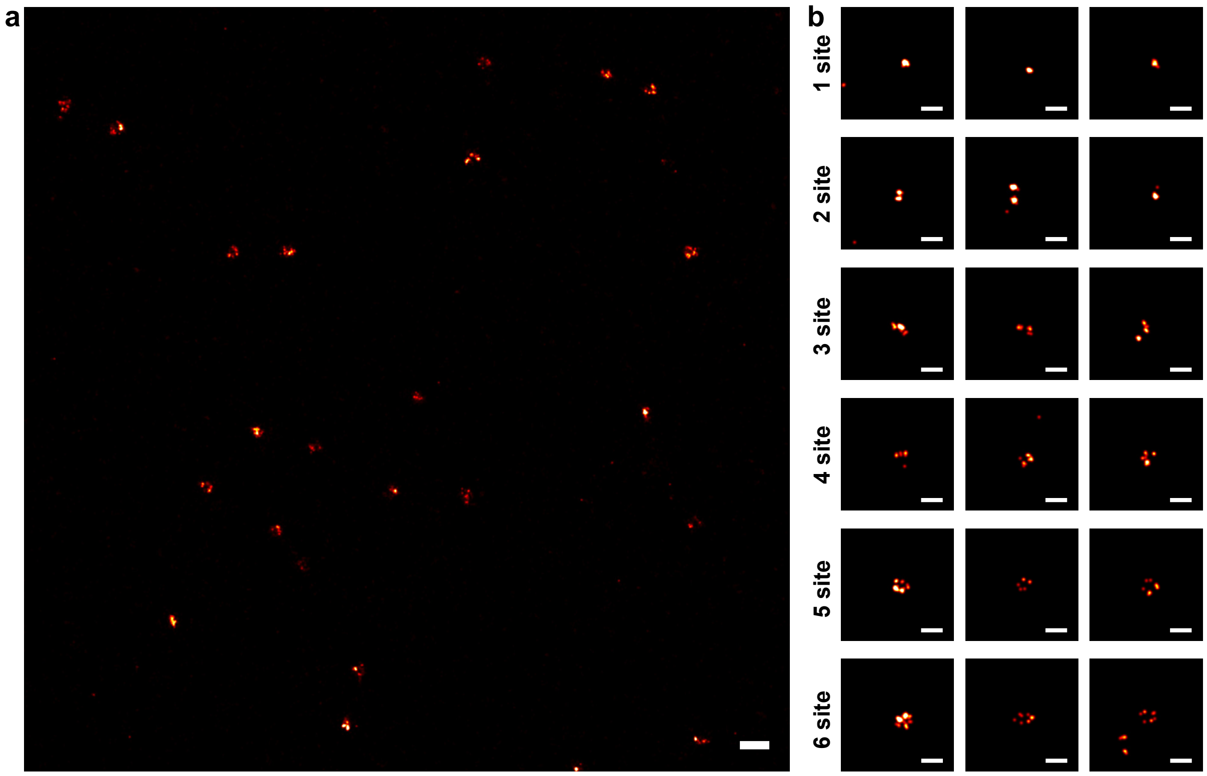

Figure S20. DNA-PAINT images of W19 structures. (a) A field of view DNA PAINT image of the W19 structures (Scale bar = 200nm). (b) Rendered images of origami ROIs containing 1, 2, 3, 4, 5 and 6 detected TRAIL peptides (Scale bar = 50nm).

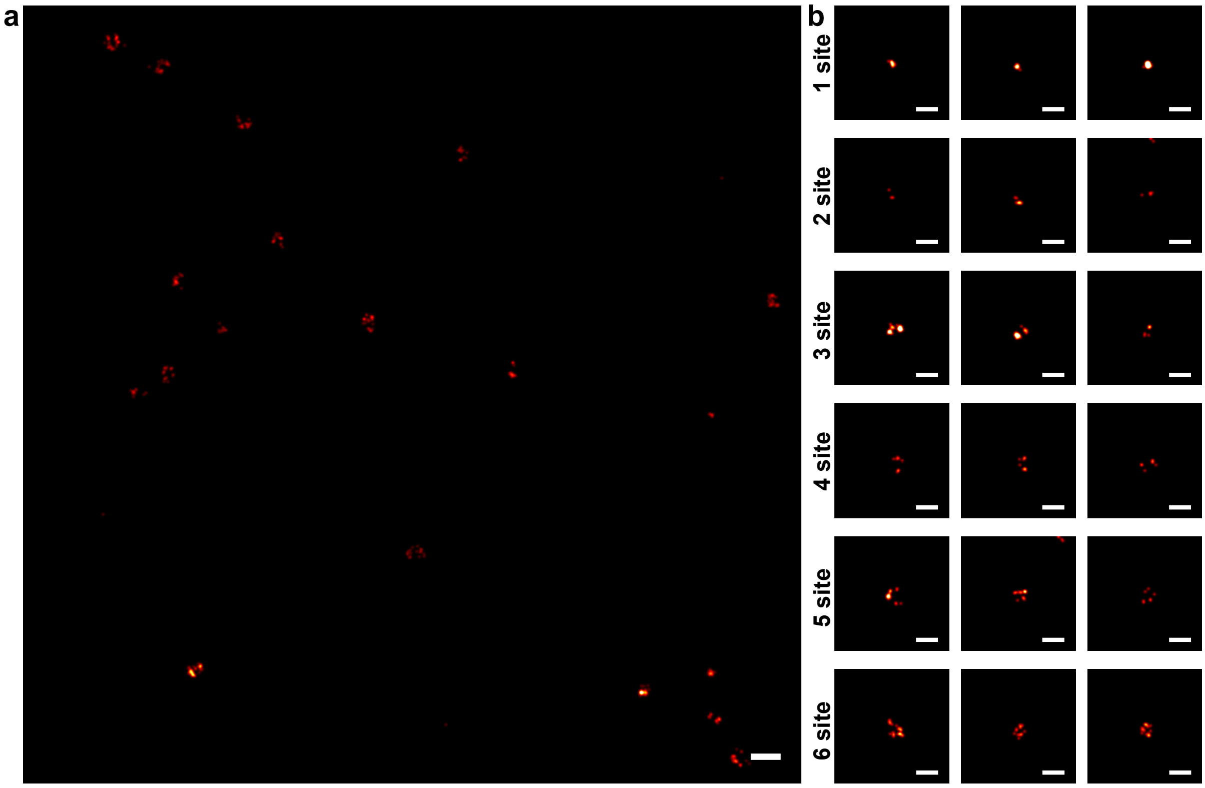

Figure S21. DNA-PAINT images of W16 structures. (a) A field of view DNA PAINT image of the W16 structures (Scale bar = 200nm). (b) Rendered images of origami ROIs containing 1, 2, 3, 4, 5 and 6 detected TRAIL peptides (Scale bar = 50nm).

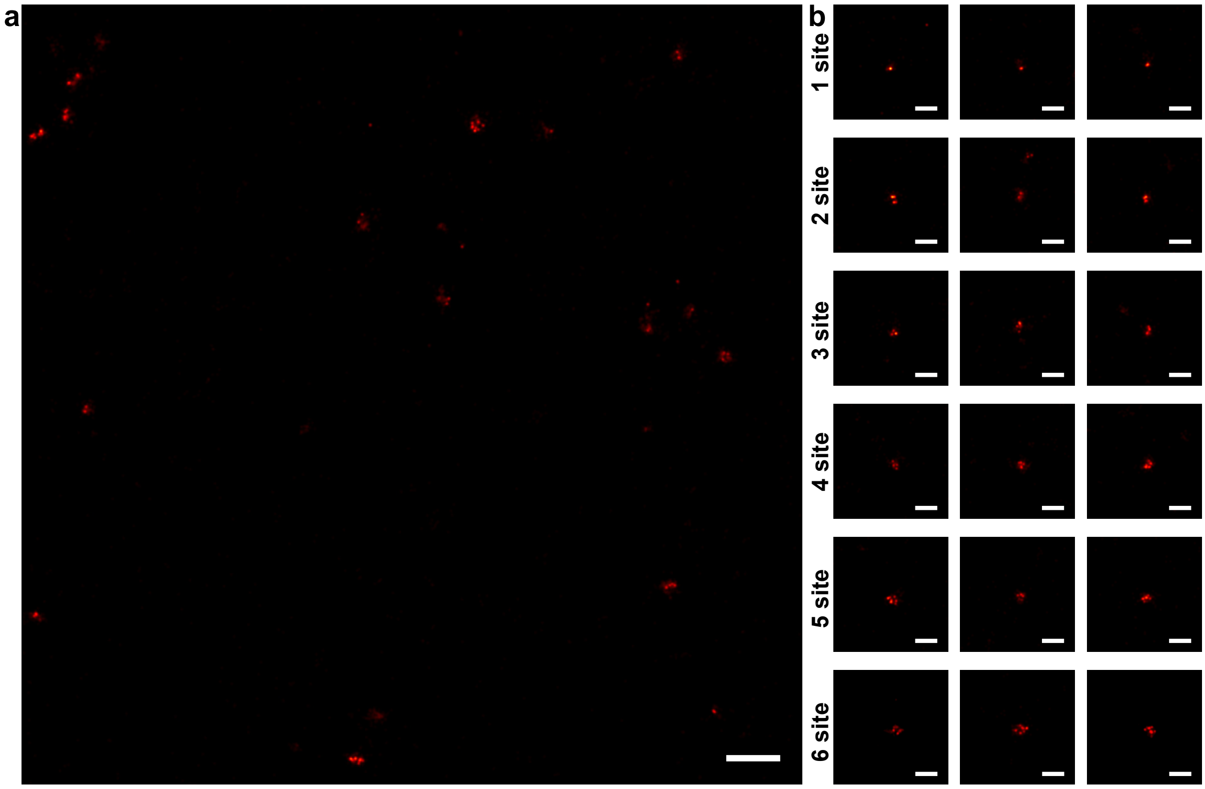

Figure S22| DNA-PAINT images of W9 structures. (a) A field of view DNA PAINT image of the W9 structures (Scale bar = 200nm). (b) Rendered images of origami ROIs containing 1, 2, 3, 4, 5 and 6 detected TRAIL peptides (Scale bar = 50nm).

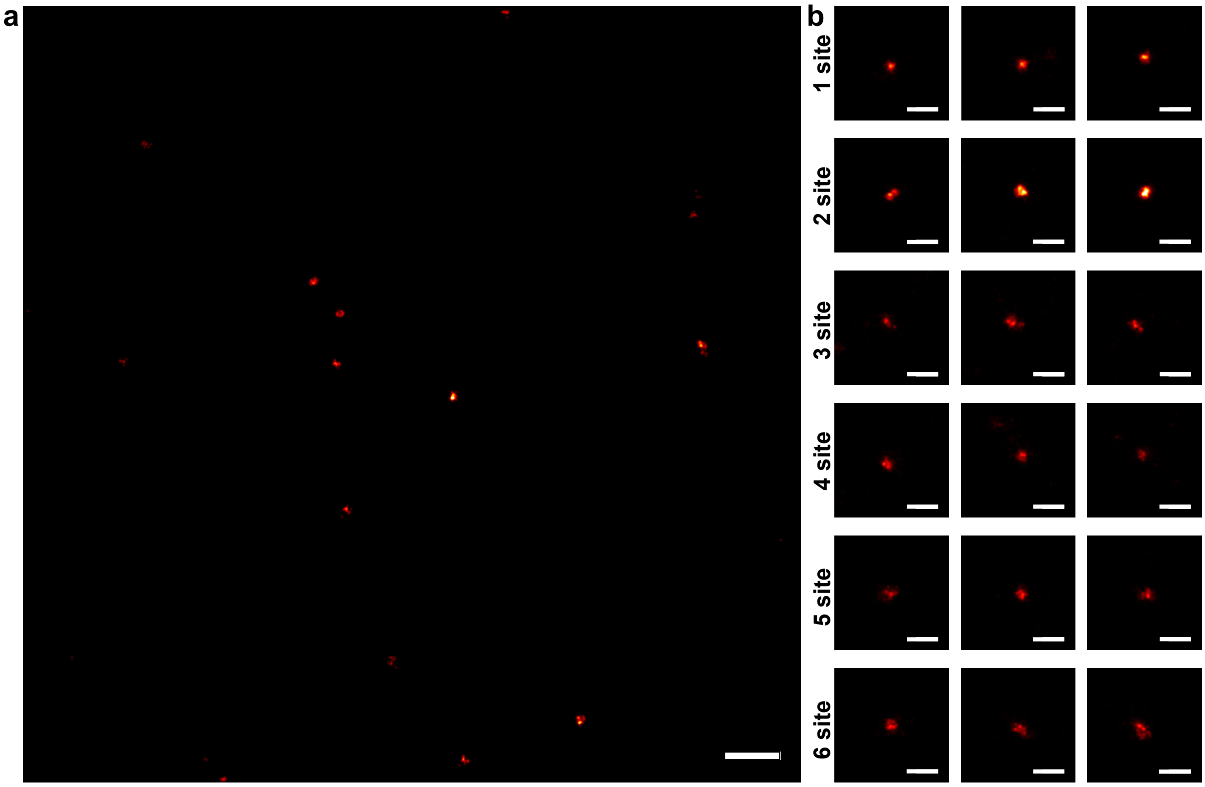

Figure S23. DNA-PAINT images of W6 structures. (a) A field of view DNA PAINT image of the W6 structures (Scale bar = 200nm). (b) Rendered images of origami ROIs containing 1, 2, 3, 4, 5 and 6 detected TRAIL peptides (Scale bar = 50nm).

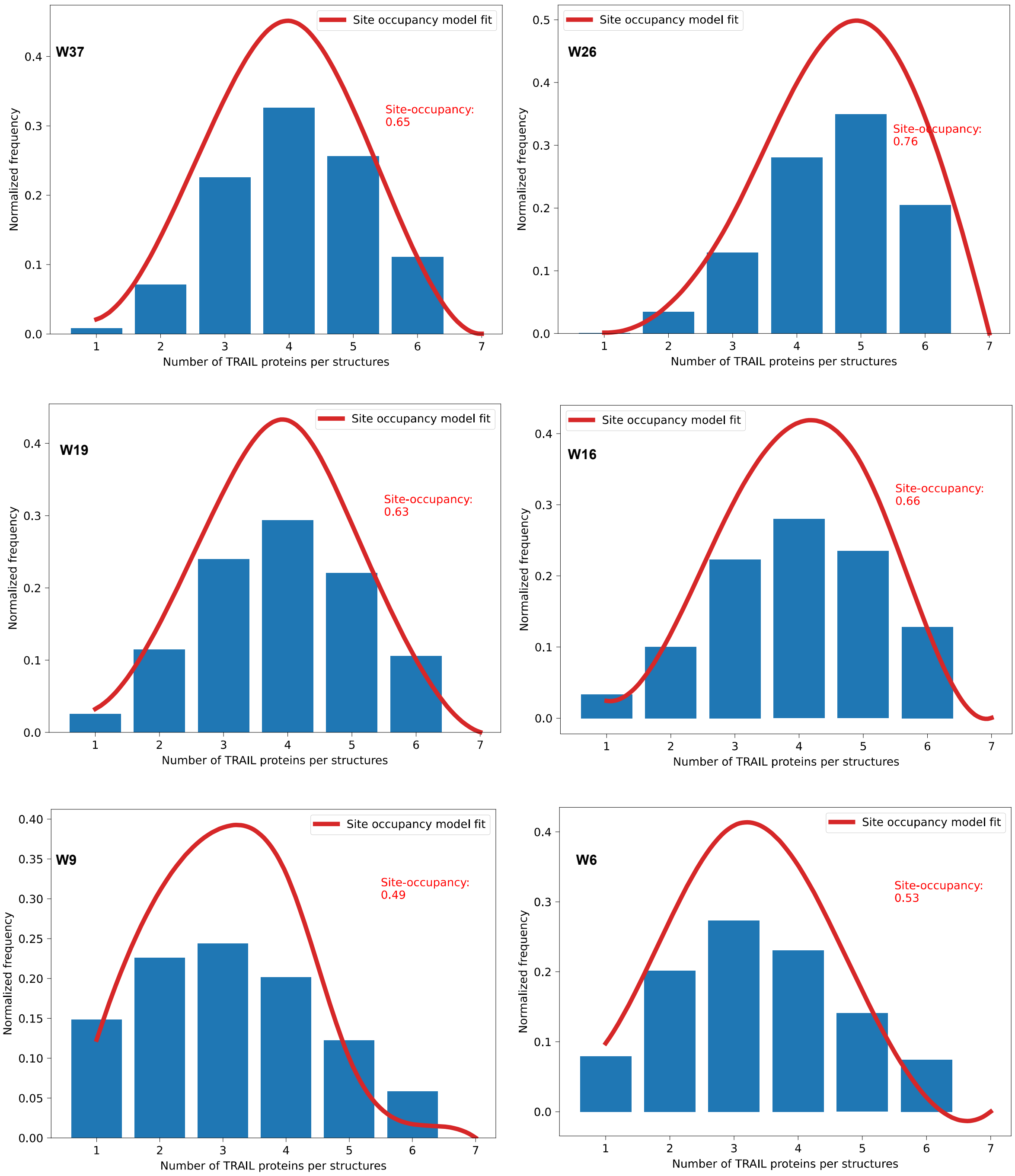

Figure S24. Protruding DNA site occupancy and the distribution of number of peptide per structure for each W-structure. This is computed from DNA-PAINT data.

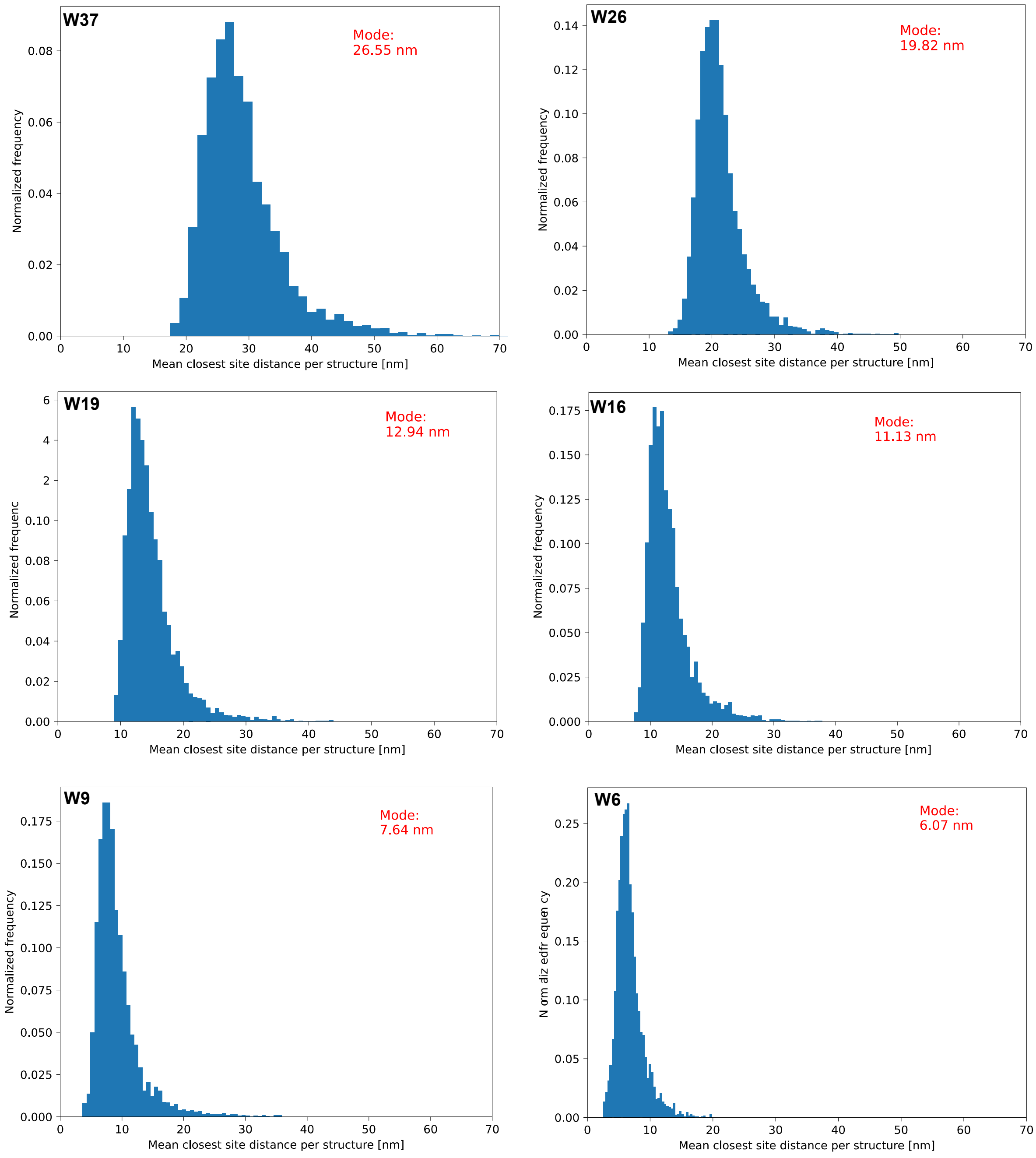

Figure S25. The inter-peptide distance distribution of each W-structure. This is computed from DNA-PAINT data.

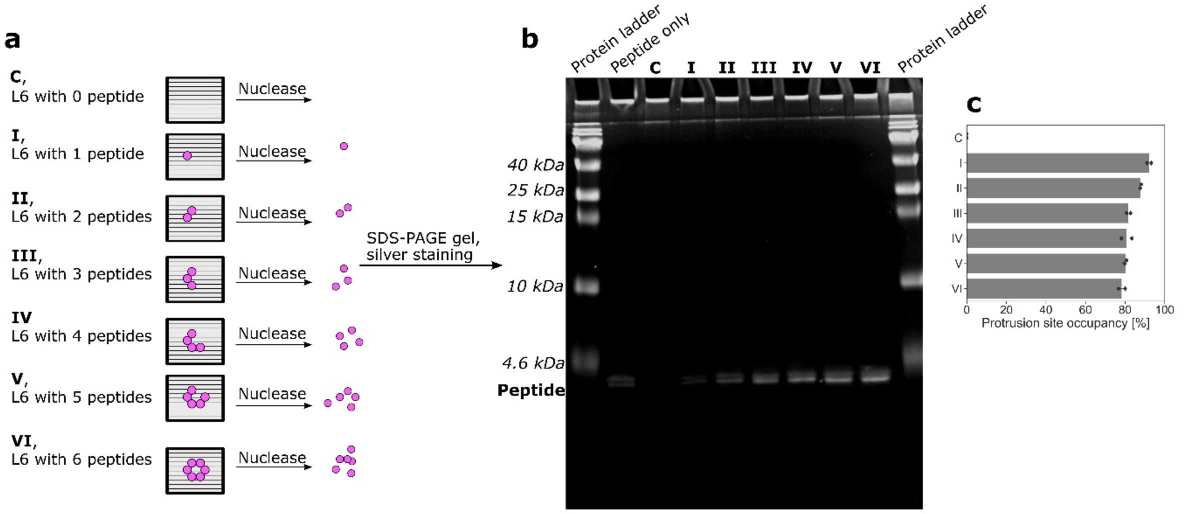

Figure S26. Gel-based peptide quantification of L6. L6 structures attached with different numbers of peptides (a) were prepared, then they were treated with DNase I to completely digest the DNA origami template. The samples were then run on SDS-PAGE (4-20% gradient gel) gel. Silver staining was carried out to visualize the peptide (b). We did not see obvious DNase I band, which could be caused by its very low loading amount. The protrusion site occupancy calculated from the band intensities plotted in (c).

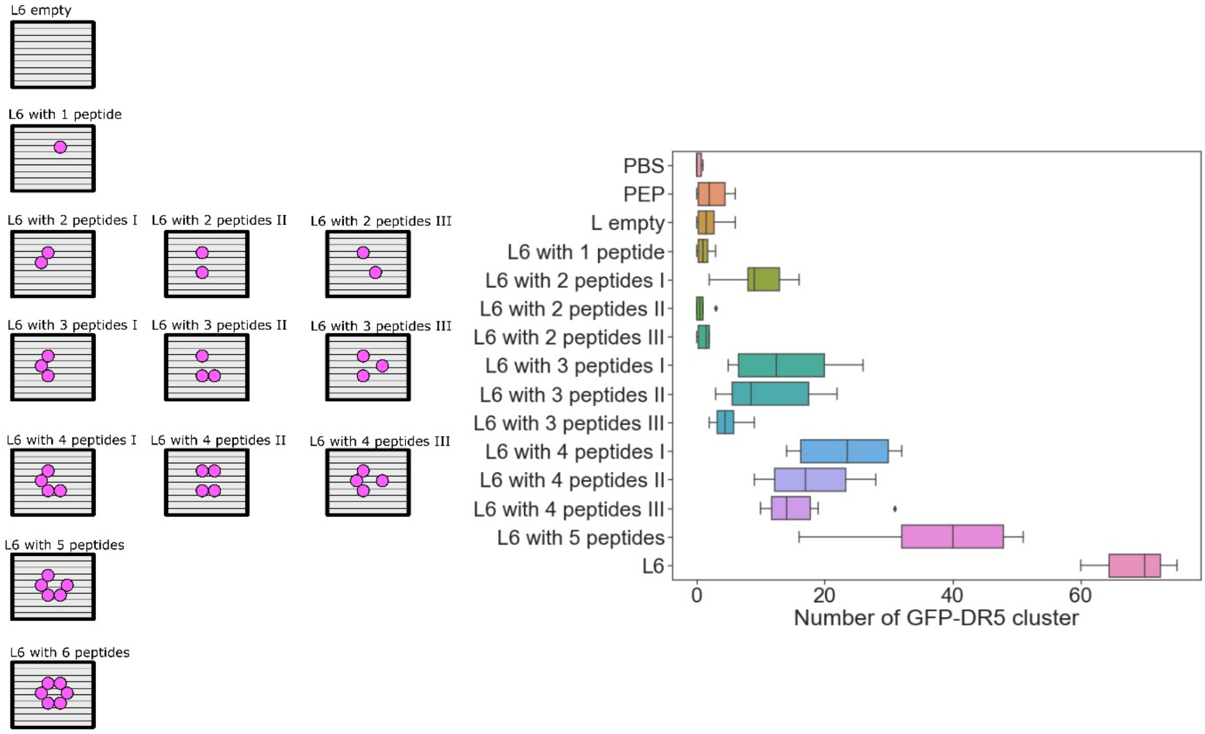

Figure S27. Quantification of GFP-DR5 clusters induced by L6 attached with different number of peptides on GFP-DR5-expressing MCF-7 cell. Left: the illustration of peptide numbers and patterns; Right: GFP-DR5 cluster counting. (n = 50 cells). The treatment was 4 hours with 2-nM DNA origami structures (12-nM peptides).

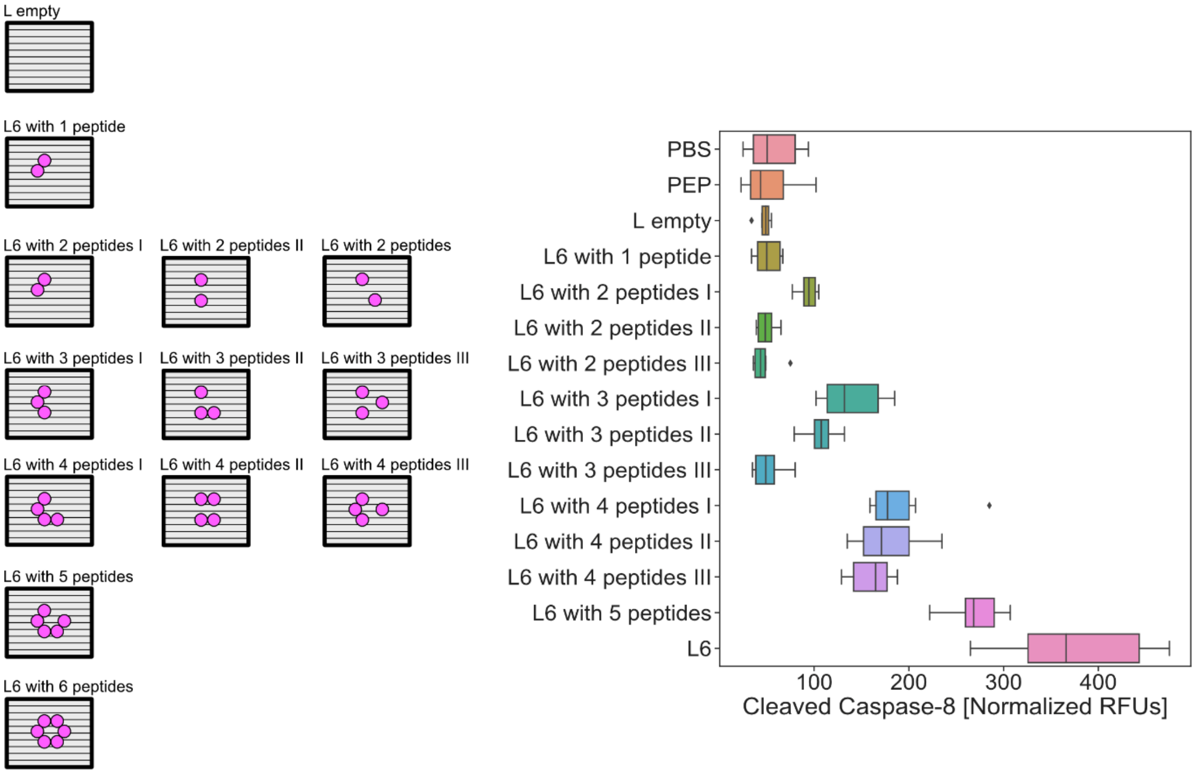

Figure S28. Normalized (to GAPDH) cleaved caspase-8 level induced by L6 attached with different number of peptides on GFP-DR5-expressing MCF-7 cell. Left: the illustration of peptide numbers and patterns; Right: Normalized (to GAPDH) cleaved caspase-8 level. The treatment was 1.5 hours with 2-nM DNA origami structures (12-nM peptides).

Extended Table.1| The half maximal inhibitory concentration (IC_{50}) of peptides.

Treatment	IC_{50} [nM] MCF-7	IC_{50} [nM] SK-BR-3	IC_{50} [nM] MDA-MB-231
Peptide	_[a]	_[a]	_[a]
W	_[b]	_[b]	_[b]
W6	14.3	9.0	9.4
W9	52.7	44.2	68.3
W16	_[a]	_[a]	_[a]
W19	_[a]	_[a]	_[a]
W26	_[a]	_[a]	_[a]
L	_[b]	_[b]	_[b]
L6	2.2	4.5	3.7
L11	_[b]	_[b]	292.7

[a] Determined up to 300 nM of peptide.

[b] Determined up to 50 nM of DNA origami structures.
